# Zhuque Base for Martian Habitation: Conceptual Design and Performance Analysis of Cave Dwellings and In Situ Construction

**DOI:** 10.34133/research.0849

**Published:** 2025-09-11

**Authors:** Cheng Zhou, Shanshan Cheng, Yuyue Gao, Jiannan Zhao, Long Xiao, Lieyun Ding

**Affiliations:** ^1^ National Center of Technology Innovation for Digital Construction, Huazhong University of Science and Technology, Wuhan 430074, Hubei, China.; ^2^ School of Civil and Hydraulic Engineering, Huazhong University of Science and Technology, Wuhan 430074, Hubei, China.; ^3^ Hubei Key Laboratory of Planetary Geology and Deep Space Exploration, Planetary Science Institute, School of Earth Sciences, China University of Geosciences, Wuhan 430074, Hubei, China.; ^4^ State Key Laboratory of Lunar and Planetary Science, Macau University of Science and Technology, Macao, China.

## Abstract

Sustainable habitation systems are critical to enabling deep-space exploration on Mars, where challenges such as low gravity, extreme thermal fluctuations, and resource constraints demand advanced structural innovations. This study introduces Zhuque Base, a habitat concept inspired by terrestrial cave-dwelling principles, optimized through finite element analysis via COMSOL Multiphysics. Three arch-based configurations—the eggshell, catenary, and 2-centered arch—were systematically evaluated by parameterizing geometric variables. The study demonstrated that eggshell arches significantly outperformed 2-centered and catenary arches in mechanical properties, reducing vertical displacement of critical points such as sidewalls by 53% and 44%, respectively. In terms of thermal efficiency, the optimal catenary arch showed 5.5% and 6.7% lower heat loss than eggshell and 2-centered arches. Furthermore, the implementation of optimal parameters (span: 3.2 m, height: 1.25 or 1.45 m) limits the peak compressive stress to 195.72 to 203.38 kPa, while the cross-sectional area can be increased by 14% to maximize the available internal space. These findings establish a parameter-driven framework for in situ Mars habitat optimization, emphasizing the trade-off between mechanical robustness and thermal efficiency in extraterrestrial structural design.

## Introduction

With the rapid development of global space technology, Mars has emerged as the next major exploration target following the Moon. Due to the similarities in geological processes and geomorphological features between Mars and Earth, it is regarded as a viable candidate for future colonization. Early exploration efforts laid the groundwork for this vision: In 2013, National Aeronautics and Space Administration (NASA)’s Curiosity rover provided critical insights into Martian geology and atmospheric conditions [1], while the ExoMars Trace Gas Orbiter mission (2014) mapped surface composition [2], revealing potential resources for in situ resource utilization (ISRU). By 2015, the first conceptual designs for Mars habitats were proposed, emphasizing the need for lightweight, radiation-resistant structures [3]. These early studies established the foundational knowledge for subsequent missions, including NASA’s Mars 2020 mission [4], China’s Tianwen-1 rover [5], and the Hope rover developed by the United Arab Emirates Space Agency (UAESA) [6]. Furthermore, the ultimate goal of NASA’s Moon to Mars architecture (2021) is to achieve a crewed landing on Mars and establish a durable research station [7]. These missions not only signify a new era in Mars exploration but also provide valuable data support and technological advancements for the construction of future Mars bases.

Nevertheless, the construction of a Mars base faces many unprecedented challenges, primarily arising from the planet’s extreme environment and the high costs associated with material transportation [8,9]. The Martian atmosphere is thin, with an average surface temperature of approximately 210.15 K and a temperature variation exceeding 100 K [10,11]. Additionally, the surface radiation levels on Mars are 100 to 1,000 times higher than on Earth [12], primarily due to galactic cosmic rays and solar particle events. These high-energy particles can penetrate even thick shielding materials, causing DNA damage, increased cancer risk, and long-term neurological effects in astronauts. The expense of transporting materials from Earth to Mars is significantly greater than that to the Moon, with costs reaching up to $200,000 per kilogram for payloads, making it essential to identify more cost-effective construction solutions for Martian habitats [13]. Utilizing local resources, particularly Martian regolith, is paramount for the economic and logistical feasibility of sustainable Mars base construction. As emphasized by Korniejenko et al. [14], the strategic implementation of ISRU offers a critical pathway to reduce dependence on Earth-sourced materials and overcome the immense challenge of interplanetary transportation costs. Currently, researchers have progressively transitioned their development efforts from lunar ISRU technologies to the exploration of Martian-adapted ISRU systems [15,16].

In light of these obstacles, scholars worldwide have conducted extensive research to propose a variety of construction solutions. The predominant methods can be broadly categorized into prefabricated modular assembly [17], 3-dimensional (3D) printing [18], excavation construction [19], and flexible structure unfolding [20]. Notably, the utilization of Martian caves or subsurface spaces is emerging as a particularly promising approach [21]. For instance, the 2023 Massachusetts Institute of Technology (MIT) study demonstrated the feasibility of robotic cave exploration for habitat siting [22], while 2025 experiments by the European Space Agency (ESA) validated regolith-based insulation materials under simulated Martian conditions [23]. Although research on Mars habitats has made significant advancements, many current proposals inadequately address the planet’s distinct environmental challenges or leverage its advantageous conditions. A number of designs remain conceptually similar to lunar base approaches, which leads to a neglect of key Mars-specific characteristics, such as the valuable engineering properties of Martian regolith (especially clay minerals) and the potential benefits of its elevated terrain. Consequently, there is a lack of systematic optimization for Mars’ low gravity, extreme temperature cycles, and radiation environment, alongside insufficient utilization of readily available local resources for construction. Moreover, integrated life-cycle strategies covering deployment, construction, long-term maintenance, and future expansion are rarely considered comprehensively.

This paper therefore proposes Zhuque Base, an innovative Mars habitat concept that integrates traditional cave construction with advanced engineering. Distinct from prior work, this design leverages Martian mountainous geology and soil properties to systematically optimize structural forms and construction processes, thereby enhancing long-term stability and heat insulation efficacy under low-gravity and extreme thermal cycling conditions. Furthermore, it innovatively utilizes indigenous clay minerals to develop in situ construction materials, substantially reducing dependence on terrestrial components for the primary habitat structure. Finally, the design incorporates a comprehensive life-cycle strategy encompassing initial deployment, sustained maintenance, and scalable expansion to ensure operational sustainability. Consequently, Zhuque Base addresses identified research gaps while achieving substantial improvements in structural performance, resource utilization efficiency, and long-term survivability, offering a more adaptable and practical solution for future colonization.

## Results

### Design and construction of the Zhuque Base

**Fig. 1.**
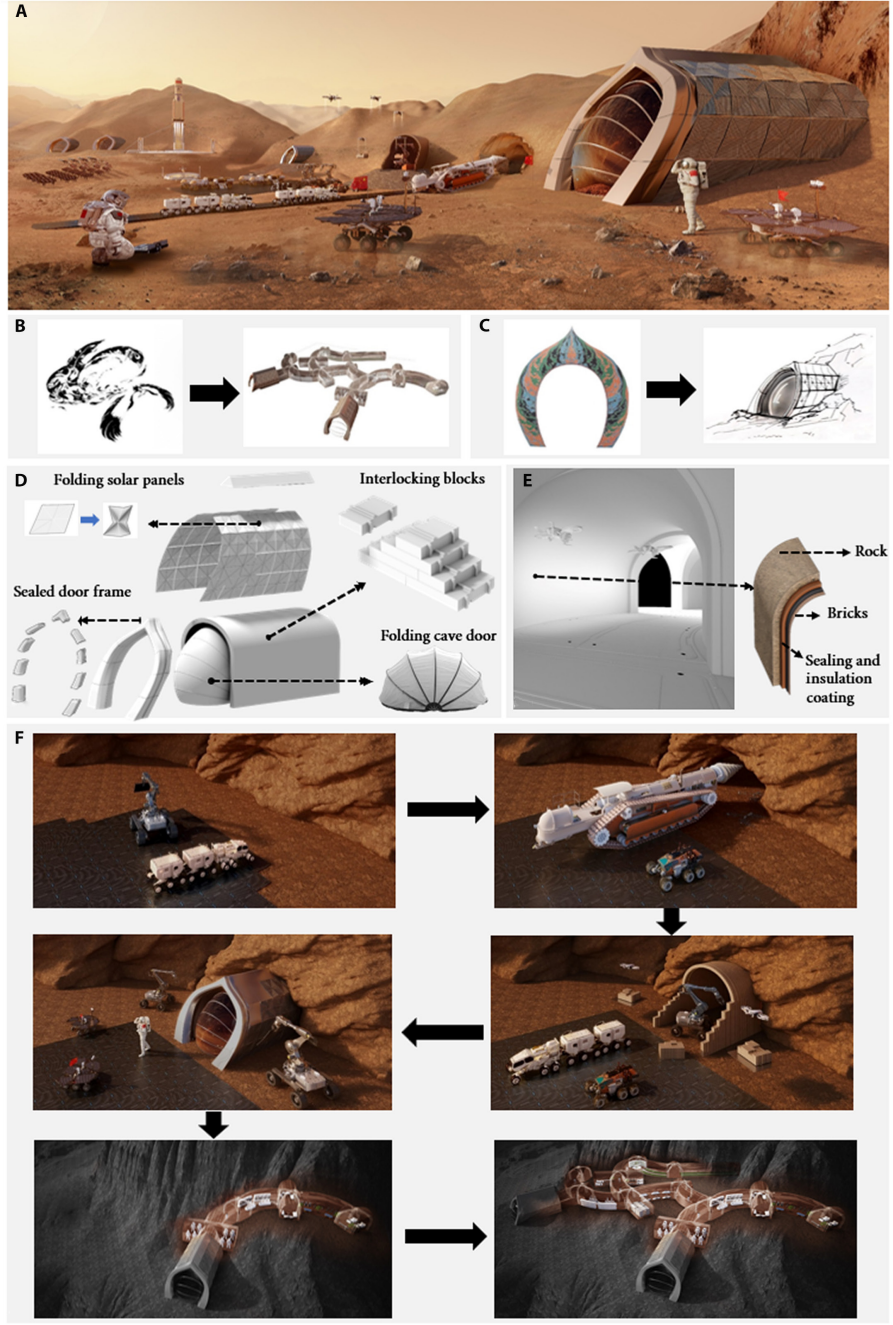
Overall conception of Zhuque Base. (A) Overall view of Zhuque Base. (B) Zhuque totem and its application. (C) Flame pattern and its application. (D) Architectural breakdown map of the entrance. (E) Architectural breakdown map of the internal arch structure. (F) Construction process of Zhuque Base.

The conceptual design of the Martian habitat (Fig. 1A) proposed in this study is inspired by one of the 4 great mythical beasts in ancient Chinese culture, known as Zhuque. The Zhuque symbolizes summer among the 4 seasons, represents the southern part of the sky, and is associated with the element of fire. Consequently, we have named the Martian habitat “Zhuque Base”. Drawing from the symbolic significance of the totem, the overall shape of the base has been designed to resemble a bird spreading its wings and soaring (Fig. 1B). Additionally, as shown in Fig. 1C, the entrance of the base features a flame-pattern design inspired by “Dunhuang” in traditional Chinese culture. This architectural form not only offers a unique visual impact but also symbolizes innovation and breakthroughs in human exploration of Mars.

The architectural design comprehensively addresses the challenges posed by Mars’ extreme environment while fulfilling the functional requirements of astronauts. Figure 1D and E illustrates the architectural breakdown of the base from both exterior and interior perspectives. In the entrance design, interlocking blocks form the main structure, integrated with highly efficient solar panels and foldable cave door assemblies to accommodate Mars’ unique conditions. The core of the base features an arch structure situated within the mountain, composed of a brick framework, sealing materials, and insulating coatings. For brick production, the decision to adopt cold pressing technology over 3D printing stems from a comprehensive evaluation across multiple criteria, including efficiency and cost of construction, structural stability, and thermal performance. Although 3D printing offers distinct advantages in rapid prototyping and the fabrication of complex geometries, cold pressing technology proves more favorable due to a precise understanding of the requirements of construction tasks and the unique characteristics of cave-dwelling structure. This method ingeniously employs mechanical pressure to compress materials into predefined shapes, eliminating the need for high-temperature sintering. The autonomous collection of regolith for this process is supported by proven robotic systems such as NASA’s RASSOR (Regolith Advanced Surface Systems Operations Robot) excavator, designed explicitly for low-gravity ISRU operations and validated in Martian regolith simulants [24]. Ishikawa [25]’s experimental validation confirms that, under Martian gravity, these pressed bricks, with a strength of 7.39 MPa, are sufficiently robust to serve as primary structural materials. Additionally, the mountain design naturally provides an outer layer of protection for the base. The compacted Martian regolith offers excellent thermal insulation and radiation resistance, ensuring the structural stability of the base and its ability to endure extreme external conditions.

At the same time, this cave-inspired construction strategy for a Mars base effectively leverages in situ resources, achieving goals of efficient, safe, and sustainable construction. The construction process can be summarized in the several key steps in Fig. 1F.

1. Initial preparation of the construction site: At the designated construction site, a comprehensive geological survey is initially conducted to identify formations suitable for caving. This phase also involves leveling the site and removing any obstructions to prepare the ground for subsequent construction activities. Meanwhile, additional shielding boxes should be installed on site, with sensitive components placed in secure, isolated locations to mitigate risks from induced electromagnetic fields [26]. When selecting construction equipment, priority should be given to those with inherent anti-electromagnetic interference designs, to guarantee stable operation amidst variations in atmospheric density, radiation levels, and induced magnetic fields caused by solar wind activity.
2. Cave excavation: Robotic excavators, such as NASA’s RASSOR, ESA’s ExoHab [27], and a swarm of mobile robots (Zebros) [28], provide the technological foundation for autonomous subsurface construction under Martian conditions. These systems have demonstrated capabilities in precision digging, regolith transport, and collaborative operations in simulated environments. In our workflow, such machinery performs excavations aligned with design blueprints, leveraging multi-robot coordination (e.g., via artificial neural tissue control) for tasks like clearing landing pads and burying of habitat modules [29].

Throughout this process, it is essential to continuously monitor the soil properties to ensure that the structural integrity of the cave remains intact [30]. For instance, techniques such as ground-penetrating radar and LiDAR are employed for the mapping of subsurface structures to facilitate real-time geotechnical assessments. Additionally, strain gauges and load cells integrated within excavation equipment enable the measurement of soil strength and density in situ. Specifically, for loose or weakly cohesive rock formations, a slower auger drilling approach is adopted to mitigate the risk of collapse. In densely compacted zones, the use of impact tools with integrated vibration dampening mechanisms minimizes structural stress, thereby enhancing operational efficiency and safety.

3. Structural reinforcement: Following the completion of cave excavation, a series of reinforcement measures are implemented to enhance the overall strength and durability of the structure. This is achieved by applying a layer of cold-pressed bricks to the inner walls, which are then coated with a composite material that serves as a sealant and thermal insulator. Subsequently, all required living and working facilities will be established. Furthermore, to mitigate the immediate risk of physical damage from micrometeoroid impacts during the vulnerable construction phase, temporary protective measures have been implemented for completed sections [31]. This includes installing lightweight protective covers or nets at crucial structural areas of the base until permanent shielding is integrated.
4. Construction of the cave entrance: For the construction of cave entrances, interlocking bricks made from Martian regolith using a cold pressing technique are utilized. Additionally, a number of solar photovoltaic panels, such as eco-friendly printed solar cells (with a power conversion efficiency of 18.26%) [32], will be installed on the exterior of the entrance for energy supply and added protection. Finally, a foldable, tent-like entrance unit will be assembled, which is designed for easy transport and assembly while offering superior sealing and dust protection features.
5. Future expansion and scaling: A significant feature of the Zhuque Base’s architectural design is its expandable, growth-like structure, which enables scalable integration of future modules. This is achieved through:

Geological adaptation: Expansion is guided by real-time geological surveys (via radar and LiDAR) to prioritize stable regions rich in resources. This ensures structural integrity while minimizing environmental disruption.

Standardized interfaces: All modules uniformly adopt the Standard Interface for Robotic Manipulation (SIROM-A) funded by the European Union. This interface supports standardized mechanical, data, electrical, and thermal transmission. Its locking mechanism allows passive docking devices to separate in a powerless state, thereby enhancing the mechanical fault tolerance of the docking interface [33].

Robotic adaptability: Robots equipped with advanced sensors and control systems can accurately identify and adapt to different module interfaces, enabling precise alignment and safe connections during construction. For example, robots can use machine vision and laser scanning technology to identify the specific dimensions and interface types of each module, and then adjust their movements and operations accordingly [34]. This ensures that the construction process remains flexible and efficient, even when handling various module types and configurations.

This strategy allows the base to evolve incrementally, from its humble beginnings as a simple habitat to a large-scale complex, while maintaining operational reliability and resource efficiency. Specifically, the Zhuque Base is not limited to mountainous regions. Geological flexibility, modular scalability, and terrain-specific optimization allow for dynamic adaptation to different Martian terrains, such as canyons and plains.

### Stability analysis under low gravity environment

**Fig. 2.**
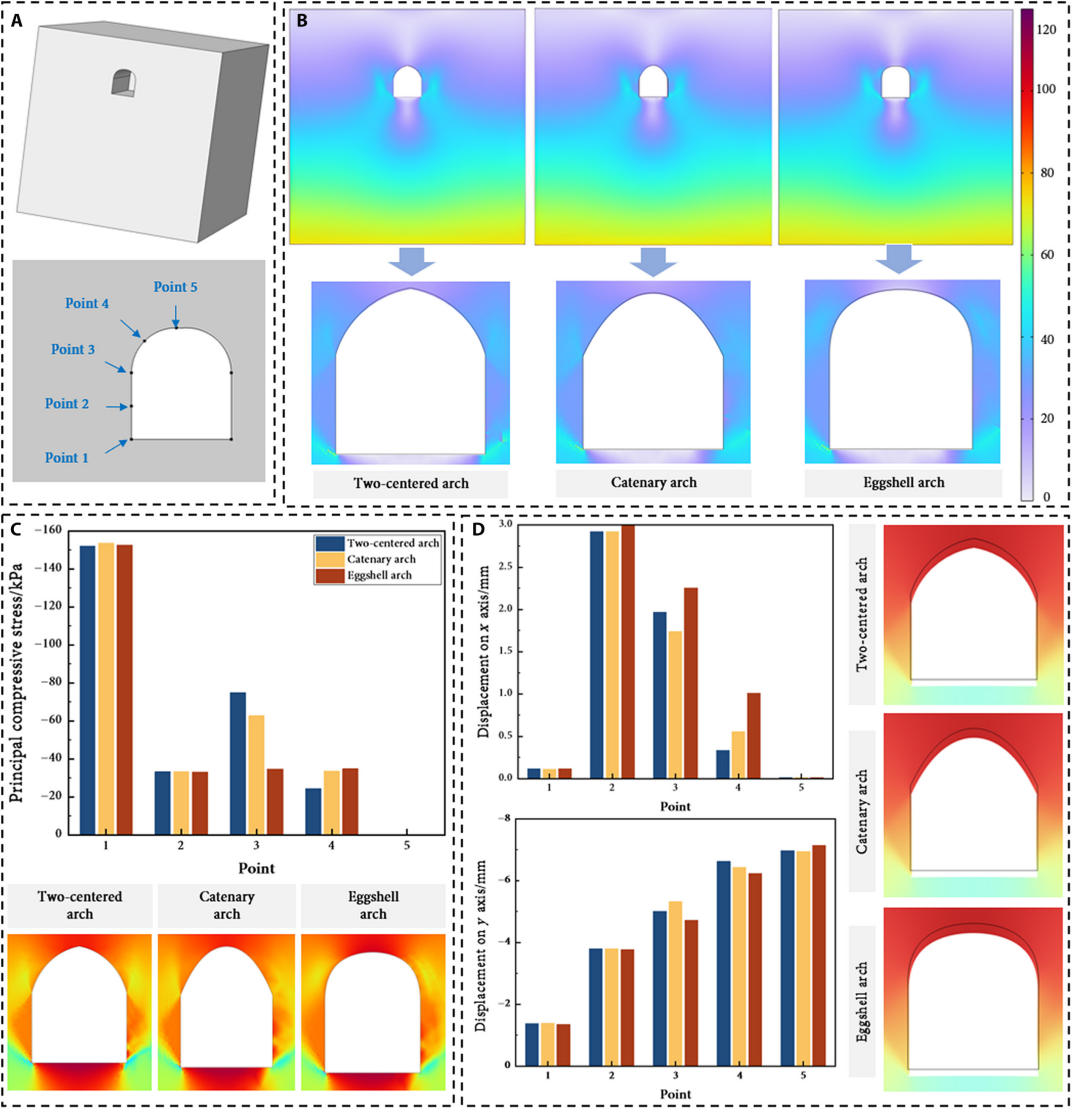
Stability analysis. (A) Schematic of the simulation model and 5 selected probe measurement points (eggshell arch as an example). (B) Von Mises stress distribution contour map. (C) Bar chart of 3 type structures and the cloud maps of minimum principal compressive stress (σ_3_). (D) Displacement on *x* axis (top), displacement on *y* axis (bottom), and the cloud maps of displacement field (magnified 10-fold).

The stress distribution in Martian habitats differs fundamentally from terrestrial structures due to the lower gravity (3.72 m/s^2^) on Mars. While 3D modeling provides a comprehensive representation, it is computationally time consuming, and multiple studies have now confirmed that 2D models can also accurately reflect the stress state of caves [35]. Furthermore, the sequential excavation methodology—entailing initial stress calculation followed by stress redistribution—may have a higher assessment of surface settlement than the actual value, leaving a safe limit for structural stability assessments [36]. Therefore, this study opts to use the 2D models for stability analysis of the 3 arch structures and selects 5 representative monitoring points on the base (Fig. 2A). The overall and detailed von Mises stress distribution of the model are illustrated in Fig. 2B. In this diagram, the *x* axis and *y* axis represent the horizontal and vertical directions of the base, respectively, while the *z* axis indicates the longitudinal depth direction of the base.

To facilitate a comparative analysis of the compressive loads among these 3 Zhuque Bases, a bar chart was produced, with specific details of the minimum principal compressive stress (σ₃) shown in Fig. 2C. Based on these diagrams, the following conclusions can be drawn:

1. The level of principal compressive stress in the soil is significantly higher at the same horizontal elevation near the base vault and footing compared to the soil located farther from the base area. The self-weight of the soil overlying the base was effectively transferred to the sidewalls via the arch structure. For all 3 Mars bases, the principal compressive stresses in the sidewalls were generally greater than in other areas of the base. Notably, a maximum value of approximately 150 kPa was observed at the root of the sidewalls. Furthermore, the principal compressive stress demonstrated a decreasing trend from the footing to the apex of the vault. At the highest point of the structure, the principal compressive stress approached 0 kPa for all 3 distinct arches.
2. The comparative lateral analysis of stress distributions across the 3 arch structures indicates that the 2-centered arch and catenary arch exhibit significant stress mutations at the junction between the sidewalls and the vault. Specifically, the principal compressive stress values recorded were 74.95 kPa for the 2-centered arch and 62.82 kPa for the catenary arch. In contrast, the eggshell arch does not display this phenomenon, attributed to its relatively smooth connection at probe point 3.

The displacements along the *x* and *y* axes for the nodes at 5 monitoring points were computed in addition to the stress analysis. Table 1 lists the mechanical properties used in the calculations. The data in Fig. 2D indicate the following conclusions:

1. The base exhibited relatively minor displacements along the *x* axis, with the soil at the center of the sidewalls reflecting the largest *x*-axis displacement of approximately 2.9 mm. This phenomenon can be attributed to 2 factors. First, the removal of the load prompts a rebound effect in the surrounding soil, causing horizontal displacement of the sidewall soil toward the center of the base. Second, the self-weight of the soil above the base vault is transmitted to the adjacent soil through the arch structure, causing the sidewall soil to shift horizontally away from the base. The interplay of these 2 mechanisms maintains the *x*-axis displacement of the Martian habitation at an overall low level.
2. The horizontal displacement of the soil surrounding the base was significantly greater than that along the *y* axis. This is evidenced by the overall subsidence of the vaulted section of the base, where the settlement progressively increases from the footing to the vault. Notably, while the vaulted area of the eggshell arch experienced the largest vertical settlement, approximately 22.15 mm, the vertical displacements at the other monitored points showed that the eggshell arch maintained more effective control compared to the other 2 base types.

**Table 1.** Summary of the key mechanics and thermal parameters of simulation materials

Materials	Parameters	Unit	Value
Regolith	Bulk density	kg/m^3^	1,400
	Elastic modulus	MPa	57.3
	Cohesive force	Kpa	11
	Friction angle	degree	25
	Poisson’s ratio	/	0.3
	Specific heat capacity	J/kg·K	630
	Thermal conductivity	W/m·K	0.039
	Surface emissivity	/	0.95
Aerogel	Density	kg/m^3^	30
	Specific heat capacity	J/kg·K	1,080
	Thermal conductivity	W/m·K	0.008
Fiber polymer	Density	kg/m^3^	920
	Specific heat capacity	J/kg·K	1,940
	Thermal conductivity	W/m·K	0.091
Basalt	Density	kg/m^3^	2,680
	Specific heat capacity	J/kg·K	806
	Thermal conductivity	W/m·K	1.3

### Thermal insulation analysis under high-temperature variation environment

To comprehensively evaluate the impact of different structural forms on the thermal insulation performance of the Zhuque Base under characteristic Martian temperature fluctuations, this study conducted a systematic thermal sensitivity analysis. The investigation encompassed 3 representative Martian seasonal conditions: aphelion (Southern Hemisphere winter solstice, Ls = 90°), perihelion (Southern Hemisphere summer solstice, Ls = 270°), and a general time condition. The steady-state heat conduction equation was solved at 6 local times (LTs): 0, 4, 8, 12, 16, and 20 h. A constant temperature of 296.15 K was maintained at the habitat’s interior surface, and the geometric parameters of the computational domain and meshing strategy remained consistent with the previous section. Thermal properties of the materials, including thermal conductivity, specific heat capacity, and surface emissivity, are detailed in Table 1. It is worth noting that significant variations in the external Martian surface temperature with season and diurnal cycle, coupled with the dual-periodic fluctuation of solar irradiance, constitute the primary external factors driving structural heat loss.

**Fig. 3.**
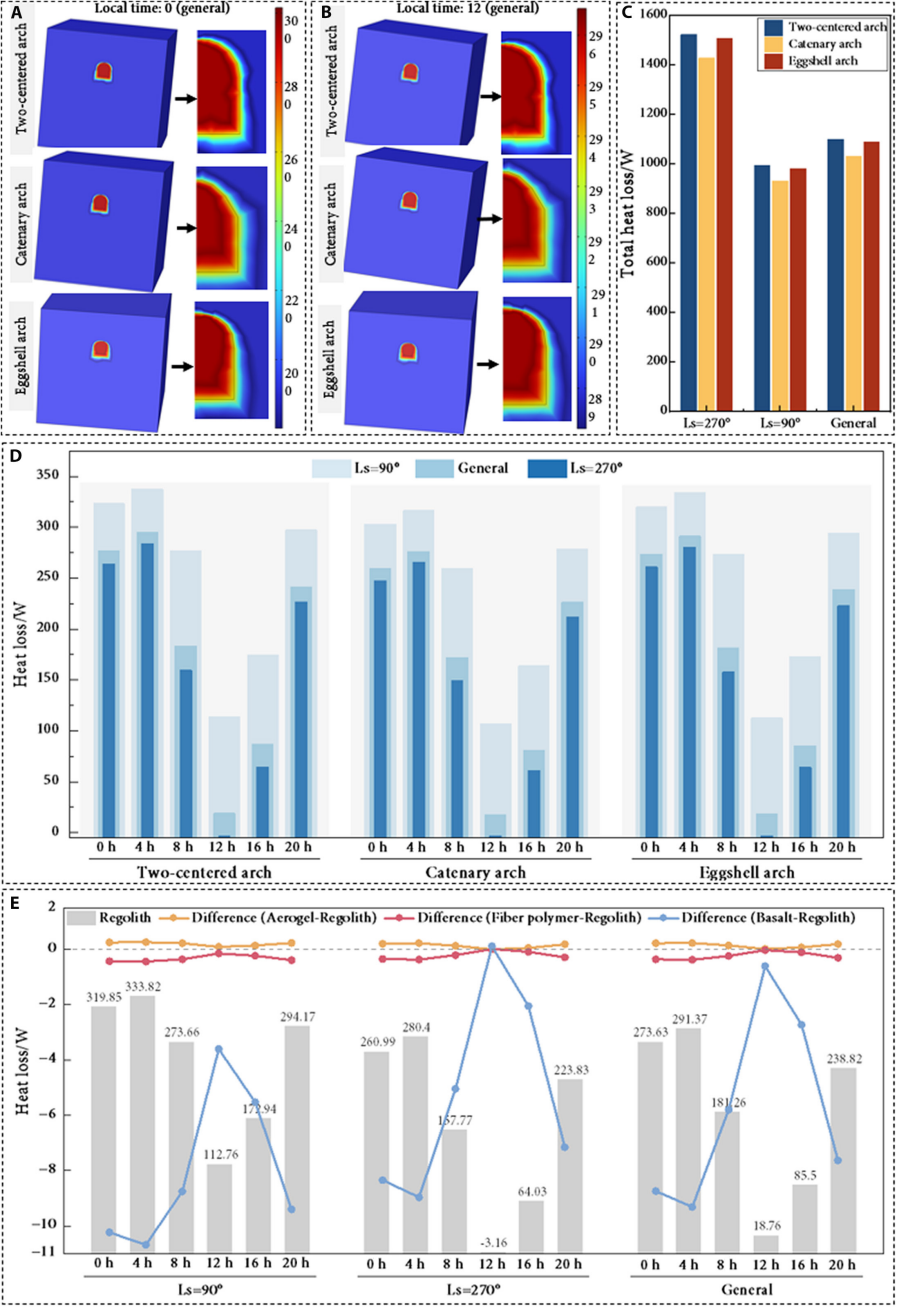
Thermal insulation analysis. Temperature field distribution for Mars noon scenario (A) and midnight scenario (B). (C) Total heat loss of 3 types of arches under 3 typical seasons. (D) Specific heat loss of 3 arches under different seasons and solar irradiance conditions. (E) Heat loss of the eggshell arch structures using different inner wall materials.

Figure 3A and B depicts the temperature field distributions for the 3 arch structures under the general time condition at 2 representative moments: Martian midnight (LT 0) and Martian noon (LT 12). Although subtle differences exist in the temperature distributions among the 3 arch types, their fundamental thermal signatures remain consistent. Notably, the spatial extent of lower-temperature zones proximate to the arch foundations exceeds that near the apices, indicating a greater tendency for heat transfer from the base interior toward the Martian surface. Moreover, Fig. 3C quantitatively summarizes the total daily heat loss for all arch configurations across the 3 seasonal conditions. The results demonstrate that the catenary arch consistently exhibits the lowest heat loss, followed by the eggshell arch, with the 2-centered arch exhibiting the highest loss. For winter solstice conditions specifically, respective thermal losses measure 1,428.5 W, 1,507.2 W, and 1,523.9 W. Relative to the catenary benchmark, the eggshell and 2-centered configurations exhibit 5.5% and 6.7% greater heat loss, respectively. This performance difference primarily stems from differences in curvature geometry and surface area-to-volume (SA/V) ratios. The catenary arch features a more naturally optimized, smooth curvature that minimizes surface area to volume ratios (SA/V), thereby reducing heat transfer pathways and decreasing overall heat loss. Its uniform curvature distribution also ensures even heat distribution, suppressing thermal gradients that could induce inefficiencies. In contrast, the increased curvature at the apex of the eggshell arch elevates the local SA/V ratio, augmenting heat loss in that region. For the 2-centered arch, the change of its curvature further aggravates the problem, making it the least efficient among the 3 designs.

Figure 3D further details the influence of solar irradiance variations across seasonal and diurnal cycles on structural heat loss. Evidently, all 3 arch configurations exhibit substantially increased heat losses throughout all diurnal phases during the transition from summer solstice to winter solstice conditions. Among different seasons, diurnal heat loss variability reaches maximum amplitude at local noon (LT 12), where winter solstice conditions produce approximately 36% and 5% greater loss compared to summer solstice and general conditions, respectively. This phenomenon primarily results from the fact that the external surface temperature at noon in the latter 2 conditions nearly approaches the internal base temperature (296.15 K), establishing reduced thermal gradients. Furthermore, within individual seasonal periods, the heat loss for all structures peaks around LT 4, with the peak loss in winter being about 19% higher than in summer. The significant drop in external surface temperature leads to an increased temperature difference, thereby driving a linear increase in heat flow. These results quantitatively reveal the amplification effect of solar irradiance and seasonal temperature differences on structural thermal load and also confirm the aforementioned annual thermal performance rankings: Eggshell arches are superior to 2-centered arches, which are inferior to catenary arches.

Building upon the established impact of arch geometry on thermal performance, this study utilized the eggshell arch configuration to investigate the potential of inner wall materials for regulating heat loss. The selected materials are all suitable for in situ construction scenarios on Mars (the key parameters are summarized in Table 1). As quantified in Fig. 3E, substitution of unprocessed regolith with fiber polymer or basalt liners reduces structural heat dissipation, whereas aerogel induces elevated thermal losses. Notably, the basalt layer exhibits superior insulating characteristics, achieving a heat loss reduction over 10 times greater than that of the fiber polymer. This thermal advantage manifests most prominently at night. For instance, under winter solstice conditions at night, using basalt as the inner wall material reduces heat loss from 333.82 W (regolith) to 323.13 W, representing a 3.2% reduction. Conversely, fiber polymer implementation yields merely 0.13% reduction in thermal loss.

In summary, this comprehensive analysis demonstrates that the catenary arch provides superior passive insulation performance due to its optimized geometry. Simultaneously, selecting specific inner wall materials, such as basalt, applied to the eggshell arch structure presents a viable complementary approach to enhance thermal regulation in Martian habitats. To address heat loss challenges, several engineering solutions could also be implemented, including strategically placing multi-layer insulation materials between the inner and outer structural layers, installing heat recovery systems within the base, and integrating phase change materials into the base walls or other structural elements.

### Selection of the optimal cross-section shape for Zhuque Base

**Fig. 4.**
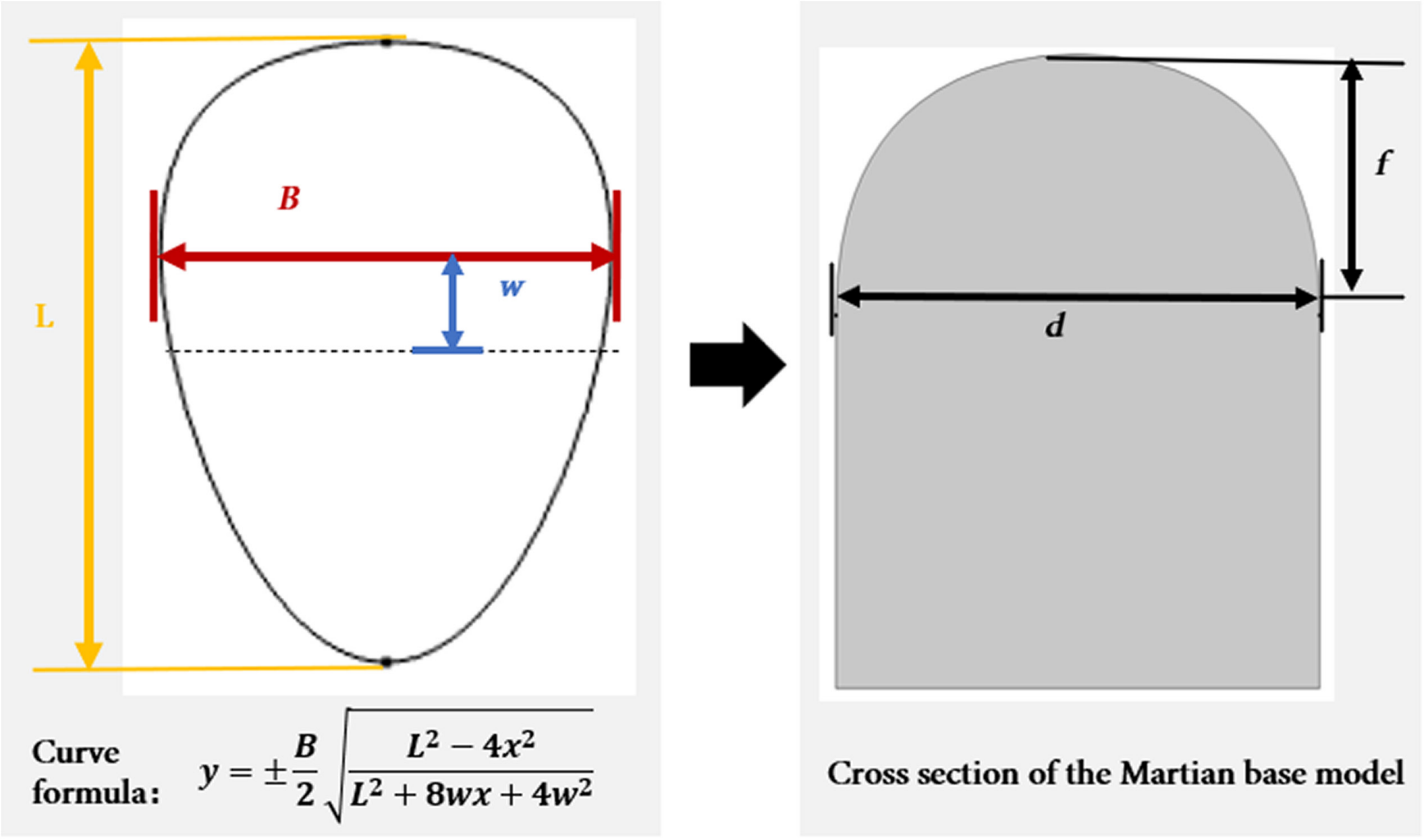
Relationship between parameters of the eggshell curve and modeling.

Following a thorough and detailed comparative analysis of the simulation results, the eggshell arch was ultimately identified as the optimal choice for Martian habitation due to its superior mechanical properties and space utilization efficiency. Although demonstrating only moderate thermal insulation performance, this deficiency is marginal and, as previously noted, can be effectively mitigated through several established engineering solutions. The preferred eggshell arch was constructed using the standard Hügelschäffer eggshell curve (Eq. 1) [37], where a direct relationship exists between the parameters in the equation and the geometric characteristics of the arch structure (Fig. 4).

The precise mathematical expression of this formula is presented as follows:

$$y=\pm \frac{B}{2}\sqrt{\frac{L^{2}-4x^{2}}{L^{2}+8\mathrm{wx}+4w^{2}}}$$ (1)

where *B* represents the maximum width of the eggshell, *L* is the total length of the eggshell, and *w* is a parameter indicating the distance between 2 vertical axes corresponding to the maximum width and half-length of the eggshell.

Obviously, a defining characteristic of the eggshell curve is that it exhibits the largest radius of curvature at its widest point. This geometry facilitates a smoother transition to the sidewall, thereby mitigating potential stress concentrations arising from abrupt connections. Consequently, the widest section of the eggshell was selected as the primary point of connection to the sidewall during the design phase. More directly, the parameter *B* directly governs the arch span (denoted as *d*), while the combination of parameters *L* and *w* determines the height of the arch (denoted as *f*).

## Discussion

In this investigation, several models with varying arch spans and heights have been constructed by controlling a single variable. Specifically, we will discuss 2 key aspects: mechanical performance and thermal insulation performance (simple scenarios, without considering the heat loss reduction scheme), while also addressing the combined effects, including the interior space factor, at the end. This study examined the effect of varying parameter *B* of the eggshell curve, which resulted in an arch span ranging from 2.8 to 3.4 m. Additionally, by modifying parameters *L* and *w*, the arch vector height of the structure was adjusted between 1.15 and 1.65 m.

**Fig. 5.**
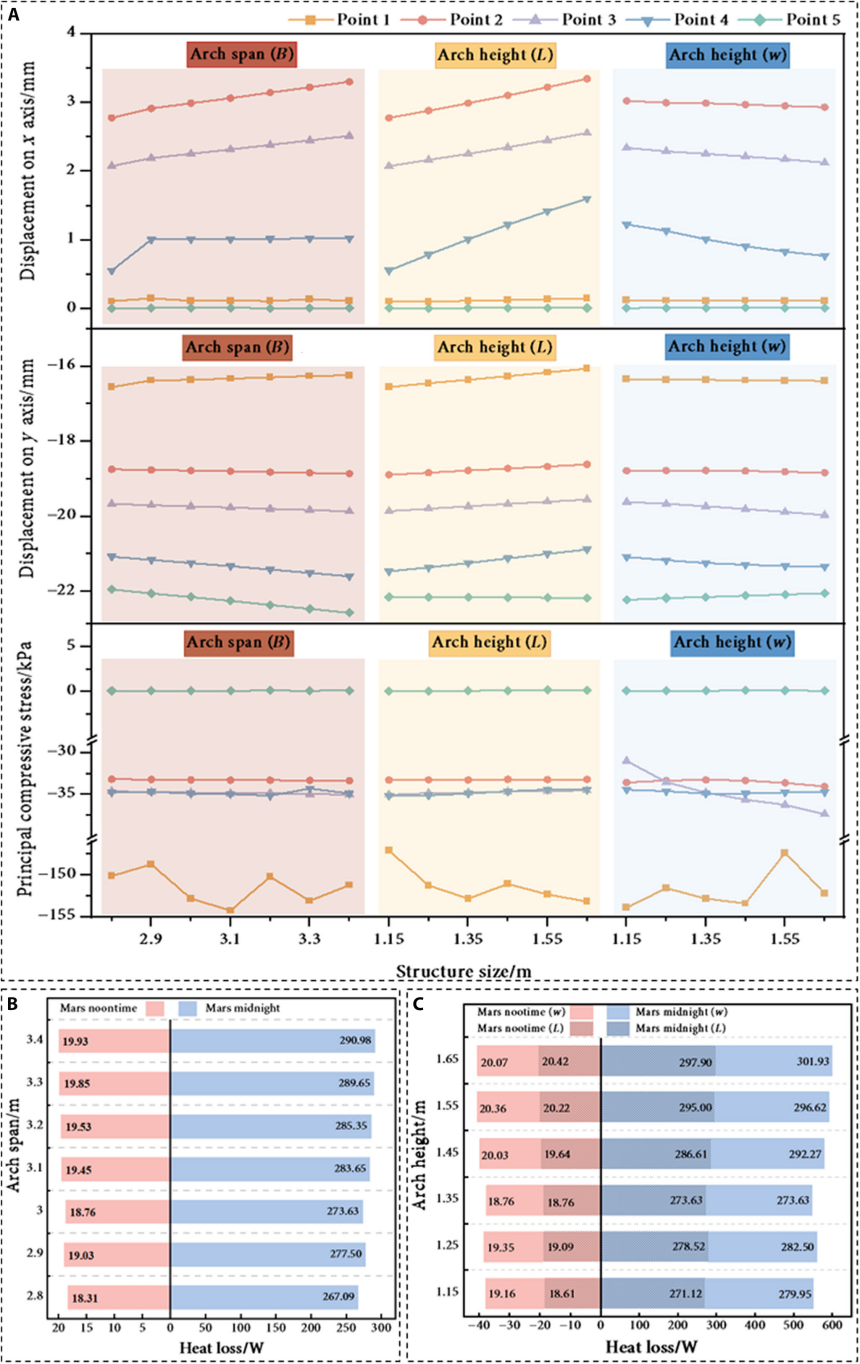
Influence of mechanical and thermal performance. (A) Effect of varying parameters (*B*, *L*, *w*) separately on the mechanical response (displacement and minimum principal compressive stress). (B and C) Effect on the thermal response (heat loss).

Initially, the mechanical stability of the structure under Martian low-gravity conditions was evaluated, with the results presented in Fig. 5A. The data presented indicate that the horizontal displacement of the mid-section of the sidewall significantly increases with a larger arch span. Similarly, increasing the parameter *L* to heighten the arch also contributes to an increase in the sidewall’s horizontal displacement, as the taller arch structure is more adept at transmitting horizontal shear forces. Conversely, adjusting the parameter *w* leads to a reduction in the horizontal displacement at the mid-section of the arch. Regarding vertical displacement, the enlargement of the arch span results in a notable rise in the vertical subsidence of the vault. In contrast, an increase in arch height causes a slight uplift of the sidewall, likely due to the taller arch’s enhanced ability to distribute the load. Furthermore, modifying the parameter *w* has minimal impact on the vertical displacement of the arch vault.

When examining principal compressive stress, it is found that the compressive stress in both the vault and the mid-section of the sidewall remains relatively stable. Nonetheless, an increase in arch span leads to a considerable rise in principal compressive stress at the base of the sidewall, which subsequently must support greater vertical loads. Additionally, increasing the parameter *L* exacerbates the stress concentration at the root of the sidewall. Notably, adjusting the parameter *w* results in an increase in principal compressive stress at the junction of the sidewall and the arch, further indicating that changes in arch curvature affect the load transfer pathways.

Similarly, the thermal insulation performance of the Mars base across the different arch span and arch height configurations discussed previously has been analyzed. The findings (Fig. 5B and C) indicate that as both the arch span and arch height increase, the heat loss from the structure during Martian noon and midnight periods exhibits an upward trend. Notably, the rate of heat loss during midnight is significantly higher than that observed at noon.

Further analysis reveals that changes in arch height have a more substantial impact on the thermal insulation performance of the structure. When adjusting the arch span, the maximum heat loss is recorded at 290.98 W. However, with modifications to the arch rise, this value increases to 297.90 and 301.93 W, respectively. Although adjusting parameter *w* significantly increases the surface area—resulting in greater heat loss compared to adjustments made to parameter *L*—the overall impact of parameter *w* on the structure’s heat loss is relatively mild. Specifically, the heat loss during the midnight period only rises by approximately 20 W and remains fairly stable throughout the adjustments to arch height. Additionally, the modification of parameter *w* results in a smoother arch curve, facilitating a more uniform temperature distribution. Consequently, the formation of localized hot spots is reduced, which in turn slows the rate of heat loss.

**Fig. 6.**
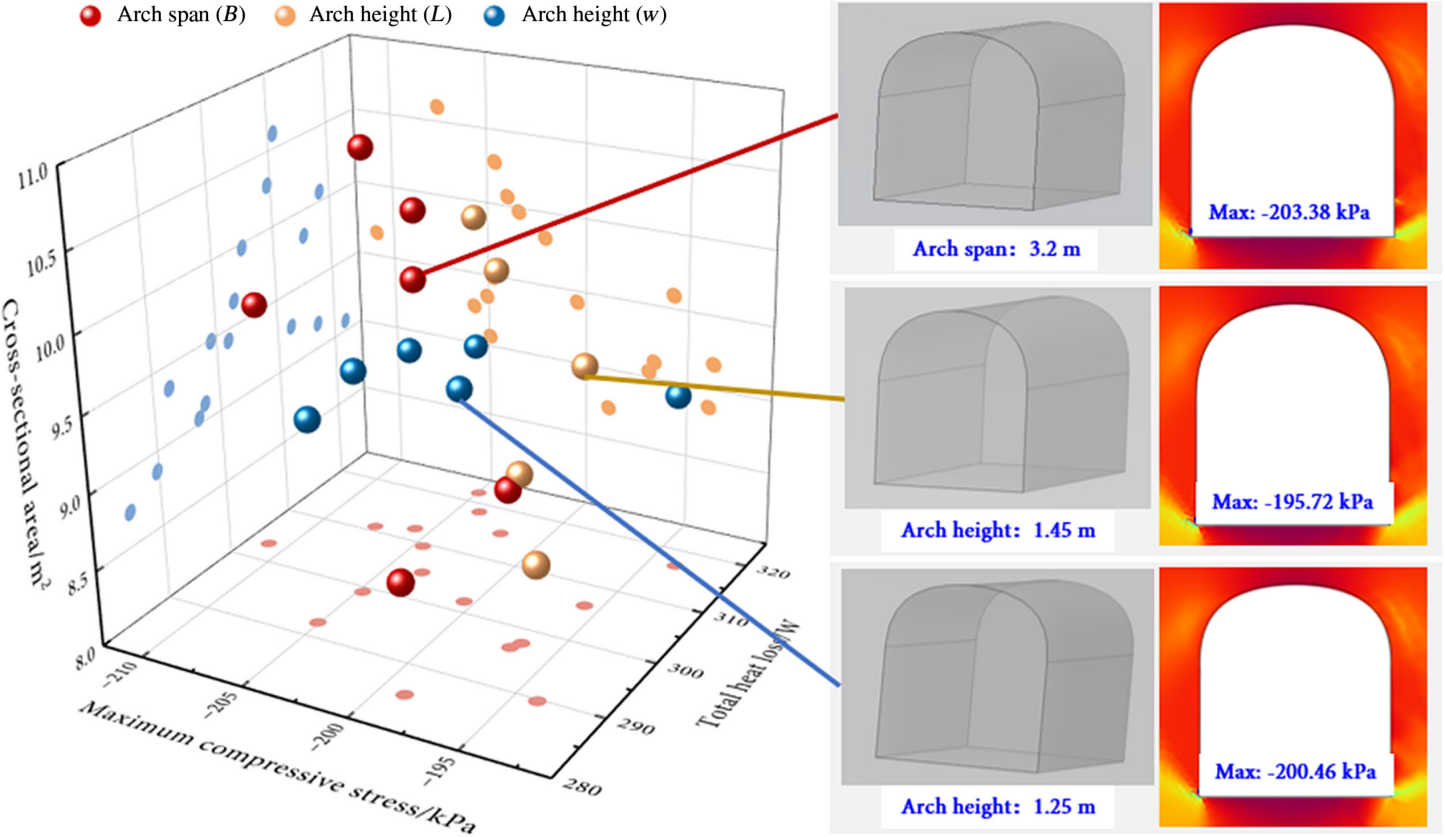
Comprehensive comparison of the effects of parameters on arch structures and relatively excellent structures.

Finally, this section provides an in-depth discussion of the impact of eggshell curve parameters on the overall performance of the structure. Theoretically, the optimal solution is achieved through the combination of parameters that minimizes the maximum compressive stress and overall heat loss of the structure while maximizing the cross-sectional area (at the same depth of entry). Table 2 summarizes the specific values of the 3 eggshell curve parameter variables of the structure and their corresponding assessment indicators, and Fig. 6 illustrates the distribution of values for the 19 sets of parameter configurations, as well as the structural models corresponding to the most efficient parameter combinations.

**Table 2.** Three sets of parameter values and the overall performance of the structure

Group	Parameter value			Maximum compressive stress/kPa	Total heat loss/W	Cross-sectional area/m^2^
	*B*	*L*	*w*			
Arch span: 2.8–3.4 m (related to *B*)	2.8	4	0.65	−198.87	285.40	8.73
	2.9	4	0.65	−196.88	296.53	9.04
	3.0	4	0.65	−205.08	292.39	9.35
	3.1	4	0.65	−210.94	303.11	9.66
	3.2	4	0.65	−203.38	304.88	9.97
	3.3	4	0.65	−204.59	309.50	10.28
	3.4	4	0.65	−207.50	310.91	10.60
Arch height: 1.15–1.65 m (related to *L*)	3.0	3.6	0.65	−193.97	289.73	8.87
	3.0	3.8	0.65	−196.63	297.61	9.11
	3.0	4.0	0.65	−205.08	292.39	9.35
	3.0	4.2	0.65	−195.72	306.25	9.59
	3.0	4.4	0.65	−202.06	315.22	9.83
	3.0	4.6	0.65	−203.96	318.32	10.06
Arch height: 1.15–1.65 m (related to *w*)	3.0	4.0	0.85	−204.79	299.11	9.49
	3.0	4.0	0.75	−200.46	301.85	9.42
	3.0	4.0	0.65	−205.080	292.39	9.35
	3.0	4.0	0.55	−205.77	312.30	9.26
	3.0	4.0	0.45	−193.77	316.98	9.17
	3.0	4.0	0.35	−204.94	322.00	9.07

From the distribution of the spheres in Fig. 6, it is evident that adjusting parameter L to change the arch height has a comparatively balanced effect on the mechanical and thermal properties of the structure. An increase in structural height results in a larger total surface area, thereby promoting heat dissipation through the surface; however, it also disperses internal stresses, leading to a reduction in maximum compressive stress. As a result, when the arch height is set at 1.25 m, the structure achieves an equilibrium point in mechanical and thermal performance, with a maximum compressive stress of 200.46 kPa.

In contrast, modifying the arch span by adjusting parameter B primarily influences the mechanical stability of the structure. A larger arch span increases the lateral support requirements, thereby impacting mechanical stability. When the arch span is set to 3.2 m, the structure demonstrates a balanced mechanical performance without significantly increasing complexity and material requirements.

The variation in the parameter *w* significantly influences the thermal insulation performance of the structure. A smaller value of *w* allows for a smoother arch curve, reducing internal airflow turbulence, promoting a more uniform temperature distribution, and minimizing the formation of local hot spots. Consequently, when the arch height is set at 1.45 m, the structure demonstrates excellent thermal insulation performance, with the maximum compressive stress recorded at just 195.72 kPa, the lowest among all cases.

## Conclusion

This study presents a conceptual design and construction method for a Martian habitation, referred to as Zhuque Base, inspired by cave dwellings. Several 2D and 3D structural models of the base were created using COMSOL, allowing for an in-depth analysis of their stability under low gravity conditions and thermal insulation performance under the considerable temperature fluctuations on Mars. Key quantitative findings and comparisons with alternative arch designs are summarized as follows:

The eggshell arch structure emerged as an optimal choice for the Mars base design, showing superior control of vertical displacement at monitoring points distinct from the vault. Results showed that the eggshell arch reduced the vertical displacement of critical points (such as sidewalls) by 53% and 44% compared to the 2-centered and catenary arch, respectively. In terms of thermal insulation performance, the eggshell arch ranks just behind the catenary arch but outperforms the 2-centered arch.

The adjustment of the arch span significantly impacts the mechanical stability of the structure. Simulation results indicate that when the arch span is set to 3.2 m, the structure exhibits optimal mechanical properties. At the same time, the cross-sectional area is significantly increased by 14% compared to a span of 2.8 m.

Conversely, variations in arch height substantially influence both the mechanical and thermal properties of the base. By setting the arch height to 1.25 m through the adjustment of parameter L, an excellent balance between these properties was achieved. Furthermore, when parameter *w* was adjusted to establish the arch height at 1.45 m, the structure displayed enhanced thermal insulation performance while minimizing the principal compressive stress to 195.72 kPa.

In summary, this study establishes a quantitative framework for optimizing preliminary Martian habitat designs through integration of core environmental parameters. However, the research scope remains constrained by current technological and scientific boundaries. At present, the Mars modeling process mainly focuses on critical factors such as extreme temperature fluctuations, but more environmental variables should be incorporated to improve the accuracy and practicality of the model. In addition, operational challenges including construction logistics and habitat system sustainability necessitate deeper analysis. In terms of future research directions, the first priority is to incorporate environmental factors such as solar wind and micrometeoroid impacts into simulations to enhance the comprehensiveness of Mars environment simulations. Ultimately, targeted studies should focus on resolving practical issues related to the construction and operation of human settlements on Mars, ensuring the long-term sustainability and functionality of the living environment. These initiatives will enable transformation of conceptual habitat frameworks into operationally viable, long-term human settlements on Mars.

## Methods

### Basic design requirements for Martian base

The significant differences between Martian and Earth environments are a fundamental consideration in the construction of a Mars base. These environmental conditions not only profoundly influence the structural design but also impose stringent requirements on the construction methods employed. A comprehensive understanding of the Martian environment is thus essential for the successful design and construction of habitable structures on Mars. Table S1 provides an overview of the primary differences in environmental characteristics between Earth and Mars [38].

As the first line of defense against the external environment, the Mars Base must withstand the negative effects of radiation, sandstorms, and extreme temperature fluctuations, among other factors that could disrupt normal internal activities. The abundant regolith and basalt minerals on Martian surface possess characteristics such as radiation resistance, high specific heat capacity, and low permeability constant, providing a potential source of shielding materials for base construction [39]. Calculations show that a 1-m-thick layer of Martian regolith can reduce the total radiation dose by 41% [40]. Besides effectively blocking cosmic rays and micrometeorites, it also offers excellent thermal insulation performance [41]. Given the extreme conditions present on the surface of Mars, astronauts are likely to spend the majority of their time within the base. To ensure a comfortable environment for researchers, it is essential to maintain the internal air pressure at 52.67 kPa and to regulate the temperature to a constant 296.15 K [38,42].

**Fig. 7.**
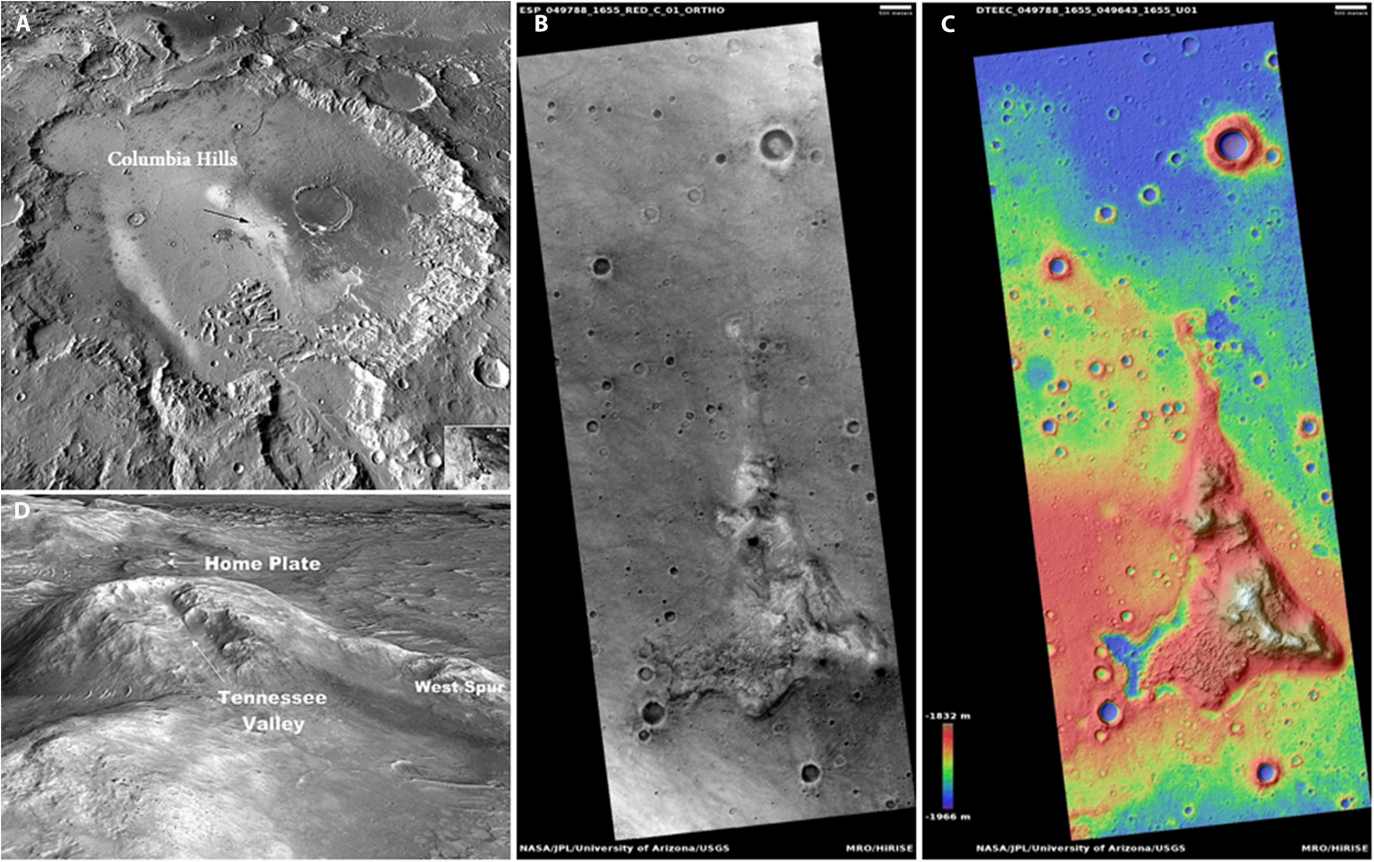
Illustration of the Martian base site. (A) Normalized THEMIS daytime thermal infrared brightness temperature mosaic of Gusev crater. (B) HiRISE image and color-coded digital elevation model map (C) of the Columbia Hills. (D) HiRISE image of the Husband Hill edifice.

In order to ensure that the base can be constructed safely, effectively utilize local resources, and achieve long-term operational sustainability, the selection of an appropriate site for a Mars Base is also a critical factor. A comprehensive site selection analysis is required, focusing on 3 key aspects: construction methods, engineering feasibility, and the availability of in situ resources. After evaluating promising alternatives such as the lava tube-rich Tharsis region and the vast canyon system of Valles Marineris [43,44], the proposed site for the Zhuque Base is Gusev Crater, located at 14.6°S, 175.4°E on Mars (Fig. 7A) [45]. This location, as the landing site of the Spirit rover, provides invaluable ground-truth data on surface properties and confirmed resources, a critical advantage over Tharsis and Valles Marineris where detailed in situ characterization and thus resource confidence remain significantly lower due to limited exploration. More specifically, the Martian base will be situated within the highest peak of the Columbia Hills, also known as “Husband Hill” (Fig. 7B).

From an engineering perspective, Husband Hill, at an elevation of approximately 107 m above the surrounding plains, offers not only the advantage of high ground but also the presence of some areas of sufficiently steep slopes to serve as natural shelters for the base [46,47]. According to available information, the landing site vicinity exhibits a low rock outcrop density (about 7 to 8% [48]) and features relatively flat terrain with average slopes between 6° and 7° [49] (Fig. 7C and D). This stands in stark contrast to the formidable challenges posed by the steep volcanic slopes prevalent across Tharsis or the deep, complex, and potentially hazardous terrain within Valles Marineris. These favorable geomorphological features at Gusev greatly reduce landing risks and simplify construction. Additionally, its low-latitude position ensures reliable solar energy potential, unlike the significant shading issues plaguing the depths of Valles Marineris or the potential complications arising from the extreme elevation of Tharsis. Compounding these advantages, favorable communication conditions exist here, whereas establishing reliable links from within Valles Marineris or mitigating potential signal effects at Tharsis’ great height would introduce significant operational complexities.

On the other hand, Gusev Crater also has its unique advantages from a natural resource perspective. A diverse range of minerals has been identified at the site, including, but not limited to, olivine, pyroxene, ilmenite, and plagioclase [50]. More importantly, while the presence of water ice has not yet been directly detected, there is substantial evidence of historical liquid water activity in the region, along with the identification of water-bearing minerals in the form of sulfates [51]. This is crucial for long-term development initiatives such as base construction and the sustainability of life-support systems at the site. This level of confirmed, accessible resource knowledge is currently unmatched in the less explored and geologically complex settings of Tharsis lava tubes or Valles Marineris, where resource presence, abundance, and extractability carry considerably higher uncertainty, posing greater risk to base self-sufficiency. Furthermore, the relative geological stability of Gusev Crater offers a more predictable environment for permanent infrastructure compared to regions like Tharsis, which displays evidence of geologically recent volcanic and tectonic activity introducing potential long-term instability hazards.

### Conceptual design of a Martian base inspired by cave dwellings

**Fig. 8.**
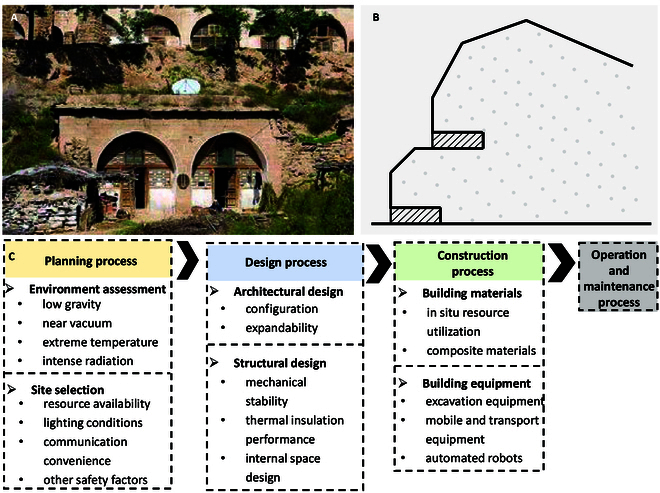
Conceptual design of a Martian base. (A) Real-life picture of cliffside cave dwellings. (B) Section drawing. (C) General process and main considerations for building a Mars base.

Referring to the requirements for base construction elaborated earlier, this paper innovatively proposes a general construction method for a Mars base inspired by the traditional cave dwellings of northwest China (Fig. 8A) [52]. This method utilizes arched caves carved into mountains as their foundational elements, effectively leveraging the existing topography while offering excellent thermal insulation. This design approach offers several advantages: it is adaptable to local conditions, the construction process is straightforward, and it promotes harmony with the Martian environment, minimizing environmental impact while providing robust protection for inhabitants.

Further, this section outlines the entire process of developing Mars bases, from initial planning to eventual operations. As shown in Fig. 8C, the approach to constructing a Mars base inspired by cave dwellings includes 4 primary steps.

The planning process for constructing a Mars base is primarily grounded in an assessment of the extreme environment and strategic site selection. Specifically, prioritizing locations with natural caves or those amenable to machine excavation can leverage the stability and protective advantages offered by Martian geology, thereby mitigating external environmental influences on the base.

The design process is central to Mars base construction and includes 2 main components: architectural design and structural design. Drawing inspiration from cave dwellings, the design approach should maximize the thermal and protective properties of Martian regolith to enhance the base’s survivability and occupant comfort.

The construction phase represents a critical stage in the overall process, emphasizing the preparation of building materials and the selection of construction equipment. Efforts should be made to source and prepare building materials from in situ resources on Mars, such as mineral, water-ice, and atmospheric resources. For example, Martian regolith can be utilized to construct the inner walls of a cave using advanced 3D printing technology.

The operation and maintenance phase is essential for the long-term stability of the Mars base. Continuous improvement and maintenance initiatives will extend the service life of the facility. The cave-inspired design of the Mars base promotes high durability and maintainability, allowing for sustained performance through regular inspections and repairs of structural wear and tear.

### Performance analysis under extreme environment on Mars

**Fig. 9.**
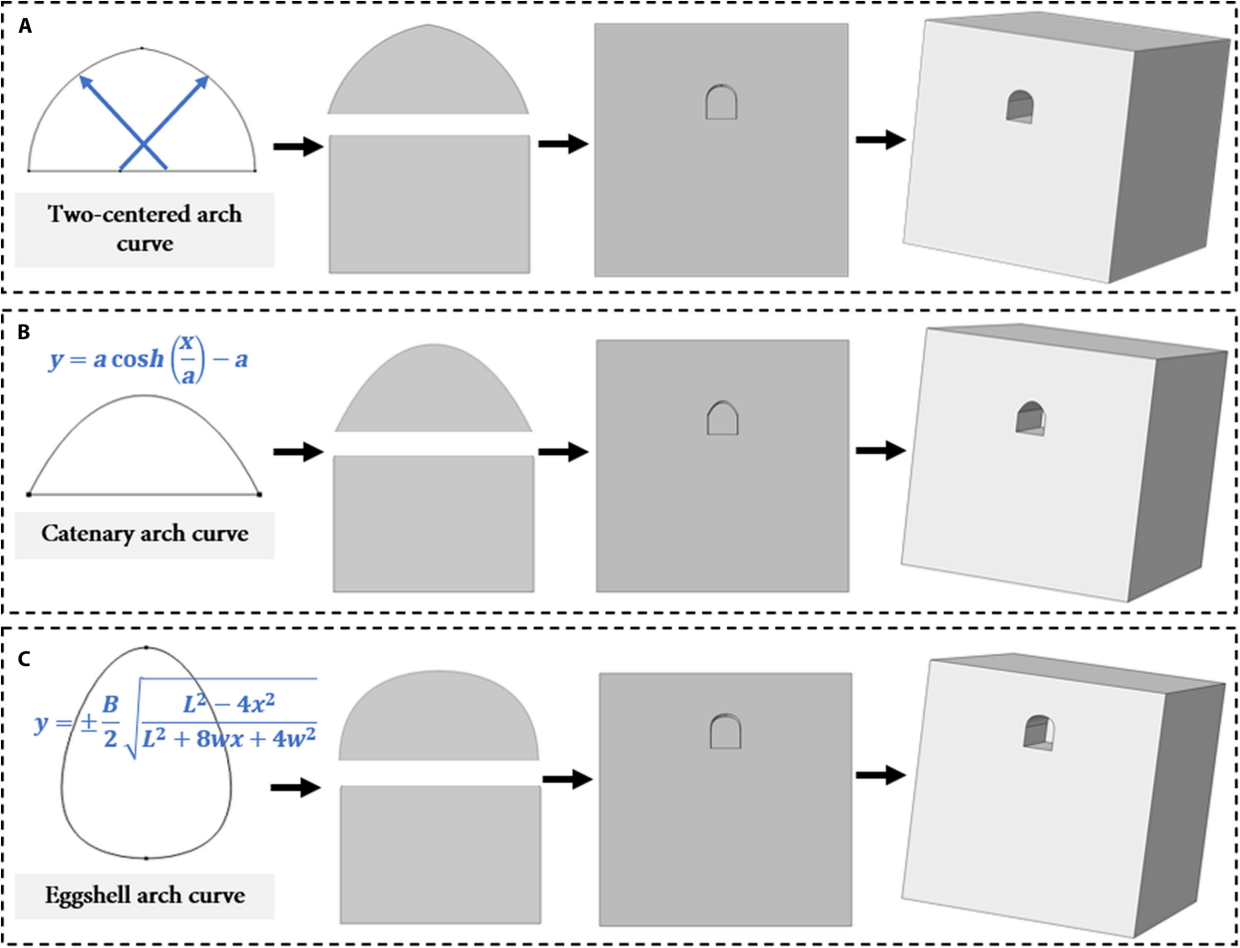
Modeling process. (A) Two-centered arch. (B) Catenary arch. (C) Eggshell arch.

This section presents the performance analysis methodology for Martian habitation inspired by cave dwelling. In this study, COMSOL was utilized to construct both 3D and 2D solid models of single-hole caves, composed of 2 main components: vertical sidewalls and a vault. To enhance the mechanical and thermal properties of the proposed Mars base, 3 distinct arch structures have been developed (Fig. 9): the 2-centered arch [53], catenary arch [54], and the eggshell arch [55].

For the purpose of subsequent comparisons, the dimensions of common cave-dwelling structures were adopted for all 3 base designs. Specifically, the arch height for each scheme is set at 1.35 m, the arch span at 3.0 m, and the sidewall height at 2.0 m. Furthermore, to closely simulate actual conditions, the thickness of the overburden at the vault is designed to be 6.0 m, with a total base depth of 8.0 m. The soil dimensions surrounding the base are established in accordance with St. Venant’s principle [56]. Accordingly, this study establishes a simulation environment that requires the soil structure surrounding the base to measure 25.0 m in width and height, with a horizontal length of 30.0 m. During the mesh division phase, careful refinement is applied particularly to the arch structure’s detailed segments.

In the analysis of mechanical properties, the mechanical loads considered are limited to the self-weight of the overburden layer resulting from Martian gravity, which is approximately 37% of Earth’s gravity. To accurately simulate the actual conditions, boundary conditions were applied to the peripheral soil body. Specifically, the normal displacements on both sides of the soil body were constrained, and the bottom was fixed as a completely rigid constraint. This study employed a 2-phase simulation approach to examine the mechanical behavior of the base under the Martian low-gravity environment. First, the displacement and stress distribution of the entire soil body under self-gravity were simulated. Subsequently, the displacement and stress changes of the base and its surrounding soil body were analyzed following an excavation that extended 8.0 m deeper into the mountain.

In the thermal insulation performance analysis, this study focuses on the 2 principal modes of heat exchange: heat conduction and heat radiation (excluding heat convection). Heat conduction refers specifically to the transfer of heat between the structure and the Martian regolith. To maintain a suitable living environment, the internal surface temperature of the structure was set to a constant 296.15 K. External surface temperature boundary conditions were obtained from Mars Climate Database v6.1 (https://www-mars.lmd.jussieu.fr/mcd_python/). Additionally, heat radiation processes include both solar and surface radiation. For the 3 arch bases studied, thermal analysis was conducted during 2 representative time periods on Mars: midnight and noon.

For solar radiation, a standard solar constant value of 1,361 W/m^2^ was utilized (as applicable to Earth’s orbit) [57]. Given that Mars orbits at a greater distance from the Sun, this value requires adjustment to accurately reflect the actual solar irradiance on Mars. The adjustment formula is provided in Eq. 2:

$$S_{\text{Mars}}=S_{0}{\left(\frac{R_{\text{Earth}}}{R_{\text{Mars}}}\right)}^{2}$$ (2)

where *S*_Mars_ and *S*_0_ represent the solar constant on Mars and at the Earth’s orbit, and *R*_Earth_ and *R*_Mars_ are the average distances from Earth and Mars to the Sun, respectively.

Furthermore, solar irradiance at a specific latitude can be calculated using the solar zenith angle. The solar irradiance received at the Martian base is determined as follows in Eq. 3:

$$I_{\mathrm{Sun}}=S_{\text{Mars}}\text{sin}\theta_{\mathrm{SZA}}$$ (3)

where $\theta_{\mathrm{SZA}}$ is the solar zenith angle and the value can be obtained from the Mars Climate Database v6.1 as well.

In regard to the irradiance reflected from Earth, the intensity of this radiation is minimal and can be considered almost negligible due to the significant distance separating Mars and Earth. The calculation for this irradiance is given by:

$$S_{\text{Earth}}=S_{0}\alpha_{\text{Earth}}{\left(\frac{R_{\text{Earth}}}{R_{\text{Mars}-\text{Earth}}}\right)}^{2}$$ (4)

where $\alpha_{\text{Earth}}$ is the albedo of the Earth and $R_{\text{Mars}-\text{Earth}}$ is the average distance from Mars to Earth.

However, the irradiance reflected from Earth to Mars is exceedingly small. Consequently, it is not included in this study, which considers solar irradiance as the primary factor influencing the thermal environment of the Mars base.

Table 1 enumerates the mechanical and thermal property parameters of the simulation materials, each with a clear cited reference source. Mechanical properties were directly adopted from the MM-2 regolith simulant experimental dataset, as its particle size distribution, loose density, and strength characteristics most closely match the physical and mechanical properties of Martian soil [58]. The dataset provides effective triaxial compression test results for elastic modulus, cohesive force, friction angle, and Poisson’s ratio. Considering that the Insight landing site (Elysium Planitia) and Columbia Hill complex are located in geologically similar volcanic regions on Mars, the relevant parameters of thermal performance can be directly referenced [59]. Therefore, with the Insight Mars mission experiment [60], the HP [3] mole was used as a modified line heat source to determine the soil thermal conductivity while setting the specific heat capacity and surface emissivity values [61]. It is noteworthy that the specific heat capacity and thermal conductivity of Martian regolith generally vary with temperature. However, the impact of temperature variation on these 2 parameters is not taken into account, as it is anticipated that their influence on the surface temperature will offset to a certain extent [62].

## Data Availability

The data are freely available upon request.
