# Supplementary material for: Zhuque Base for Martian Habitation: Conceptual Design and Performance Analysis of Cave Dwellings and In Situ Construction
Source: Research (Wash D C). 2025 Sep 11;8:0849. doi: 10.34133/research.0849 (PMC12423506; doi:10.34133/research.0849)
Supplement: Supplementary 1 — Tables S1 to S7 Movie S1 Data S1 to S9 [file research.0849.f1.zip › Supplyment Material.docx]

**Supplementary Table 1 Comparison of environmental parameters between Earth and Mars**

|  | **Earth** | **Mars** |
| --- | --- | --- |
| Acceleration of gravity/m/s^2^ | 9.81 | 3.71 |
| Pneumatic/kPa | 101.3 | 0.7 |
| Main atmospheric components | N_2_, O_2_ | CO_2_, Ar |
| Surface temperature/K | 183.95~330.05 | 130.15~308.15 |
| Solar radiation in the upper atmosphere/W/m^2^ | 1353 | 587 |
| Wind speed/m/s | average: 5 | average: 10 |
|  | max: 100 | max: 30 |
| Average annual radiation/mSv | 2.4 | 100 |

**Supplementary Video 1 Display animation of Zhuque Base (single file)**

**Supplementary Data 1 Summary of the effects of different solar irradiance and wall materials on the thermal performance (Ls=270°)**

| **Heat loss/W** | **Two-centered arch**  **(Regolith)** | **Catenary arch**  **(Regolith)** | **Eggshell arch** | | | |
| --- | --- | --- | --- | --- | --- | --- |
|  |  |  | **Regolith** | **Aerogel** | **Fiber polymer** | **Basalt** |
| Martian Day (Sol): 0 | 263.89 | 247.36 | 260.99 | 261.19 | 260.64 | 252.64 |
| Martian Day (Sol): 4 | 283.52 | 265.76 | 280.40 | 280.62 | 280.03 | 271.43 |
| Martian Day (Sol): 8 | 159.53 | 149.53 | 157.77 | 157.90 | 157.56 | 152.72 |
| Martian Day (Sol): 12 | -3.19 | -2.99 | -3.16 | -3.16 | -3.15 | -3.05 |
| Martian Day (Sol): 16 | 64.74 | 60.68 | 64.03 | 64.08 | 63.94 | 61.98 |
| Martian Day (Sol): 20 | 226.32 | 212.15 | 222.83 | 224.01 | 223.54 | 216.67 |

**Supplementary Data 2 Summary of the effects of different solar irradiance and wall materials on the thermal performance (Ls=90°)**

| **Heat loss/W** | **Two-centered arch**  **(Regolith)** | **Catenary arch**  **(Regolith)** | **Eggshell arch** | | | |
| --- | --- | --- | --- | --- | --- | --- |
|  |  |  | **Regolith** | **Aerogel** | **Fiber polymer** | **Basalt** |
| Martian Day (Sol): 0 | 323.40 | 303.15 | 319.85 | 320.10 | 319.42 | 309.61 |
| Martian Day (Sol): 4 | 337.53 | 316.39 | 333.82 | 334.08 | 333.38 | 323.13 |
| Martian Day (Sol): 8 | 276.70 | 259.37 | 273.66 | 273.88 | 273.30 | 264.90 |
| Martian Day (Sol): 12 | 114.01 | 106.87 | 112.76 | 112.85 | 112.61 | 109.15 |
| Martian Day (Sol): 16 | 174.86 | 163.91 | 172.94 | 173.08 | 172.71 | 167.41 |
| Martian Day (Sol): 20 | 297.44 | 278.81 | 294.17 | 294.40 | 293.78 | 284.76 |

**Supplementary Data 3 Summary of the effects of different solar irradiance and wall materials on the thermal performance (General)**

| **Heat loss/W** | **Two-centered arch**  **(Regolith)** | **Catenary arch**  **(Regolith)** | **Eggshell arch** | | | |
| --- | --- | --- | --- | --- | --- | --- |
|  |  |  | **Regolith** | **Aerogel** | **Fiber polymer** | **Basalt** |
| Martian Day (Sol): 0 | 276.67 | 259.35 | 273.63 | 273.85 | 273.27 | 264.88 |
| Martian Day (Sol): 4 | 294.61 | 276.16 | 291.37 | 291.60 | 290.99 | 282.05 |
| Martian Day (Sol): 8 | 183.27 | 171.80 | 181.26 | 181.40 | 181.02 | 175.46 |
| Martian Day (Sol): 12 | 18.96 | 17.78 | 18.76 | 18.77 | 18.73 | 18.15 |
| Martian Day (Sol): 16 | 86.45 | 81.04 | 85.50 | 85.57 | 85.39 | 82.77 |
| Martian Day (Sol): 20 | 241.48 | 226.35 | 238.82 | 239.01 | 238.51 | 231.18 |

**Supplementary Table 2 Summary of temperature field distributions and detailed diagrams for three arches under different solar irradiance (Ls=270°)**

|  | **Two-centered arch (Regolith)** | **Catenary arch (Regolith)** | **Eggshell arch (Regolith)** |
| --- | --- | --- | --- |
| Martian Day (Sol) 0 | 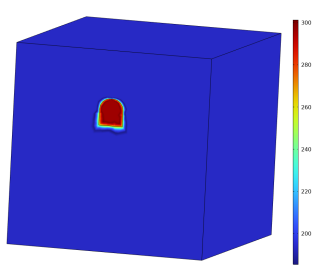 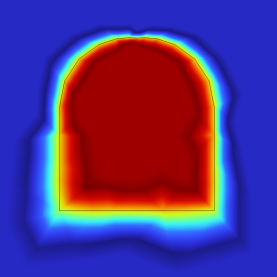 | 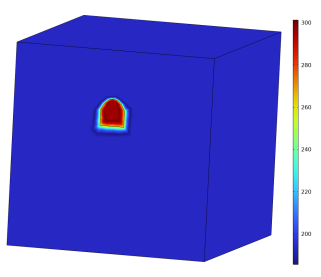 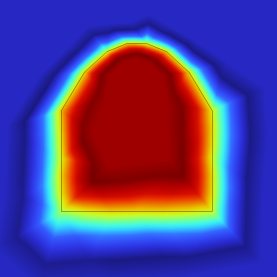 | 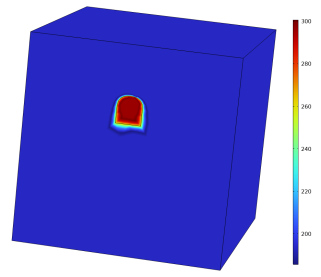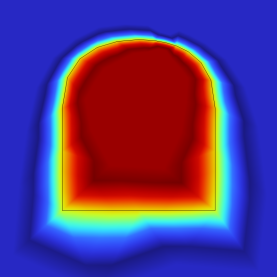 |
| Martian Day (Sol) 4 | 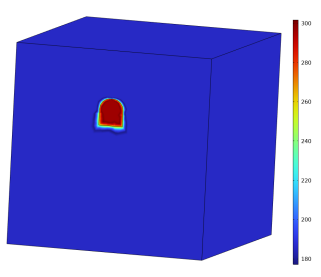 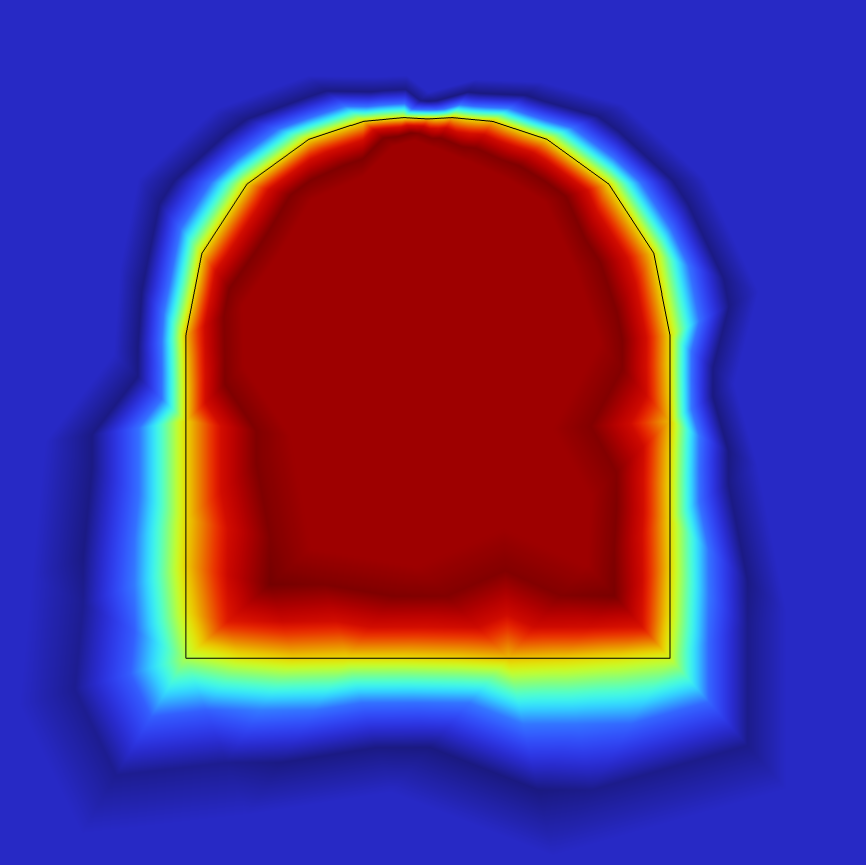 | 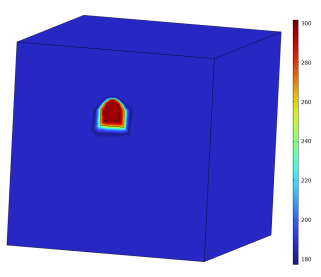 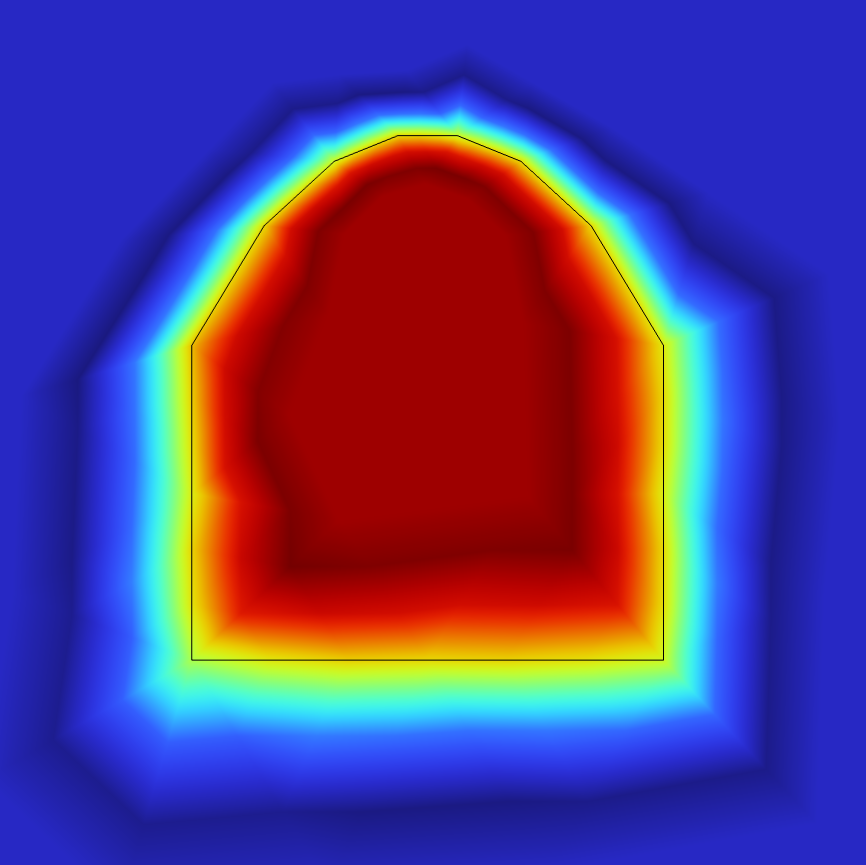 | 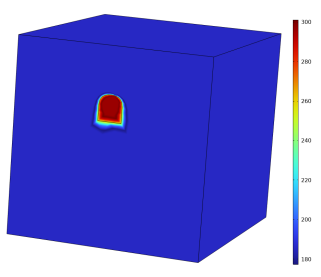 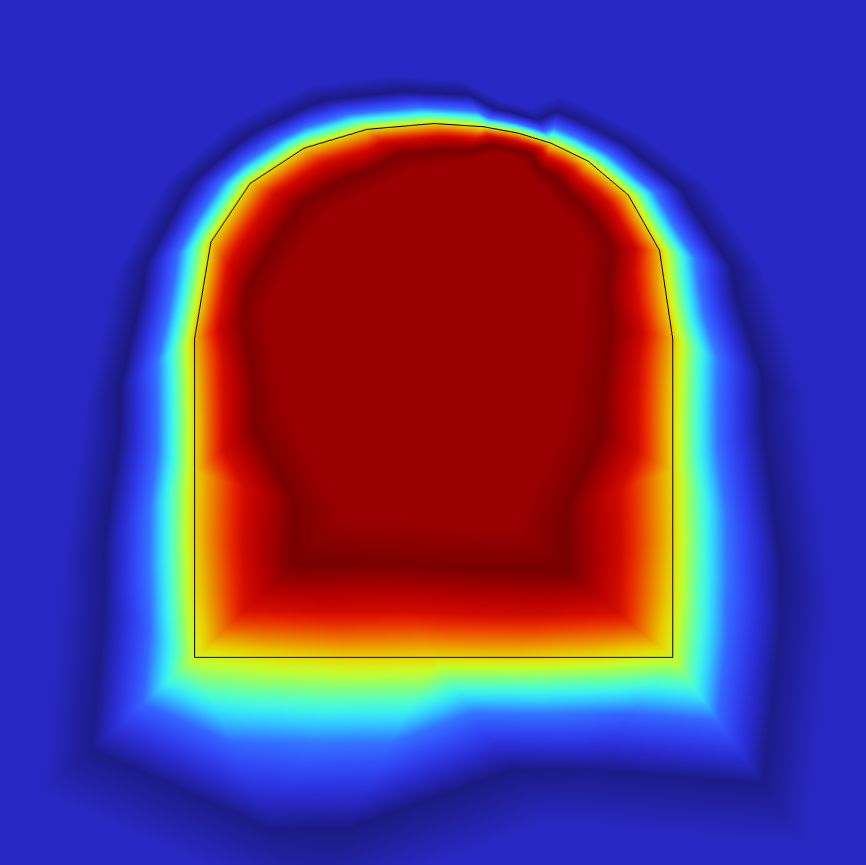 |
| Martian Day (Sol) 8 | 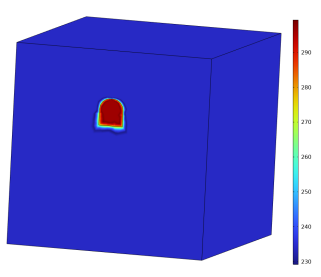 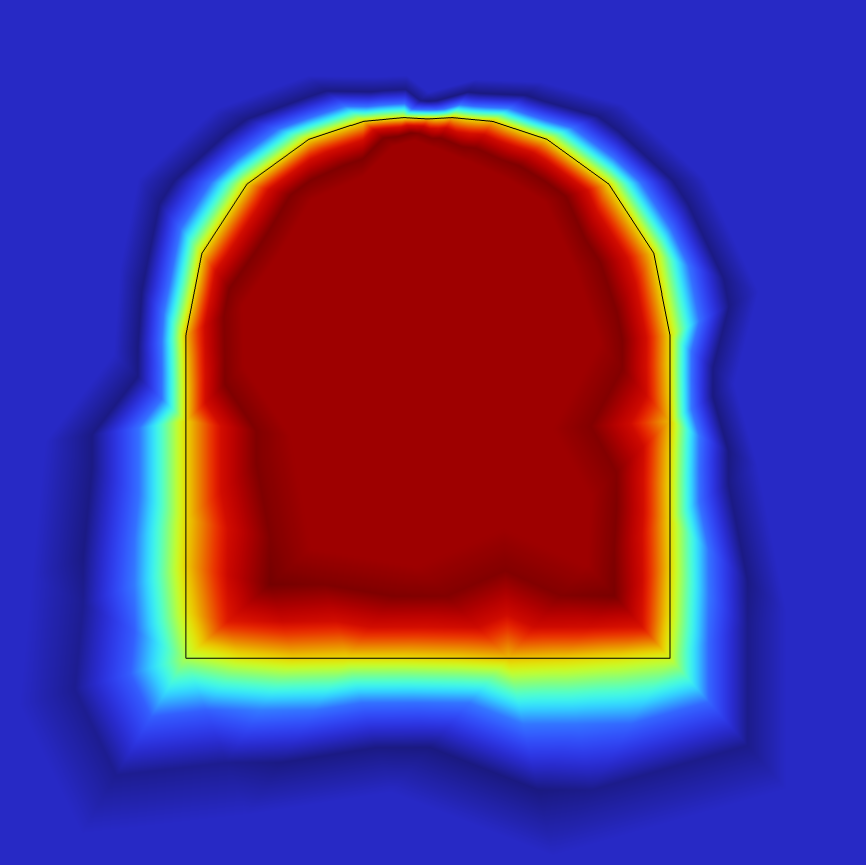 | 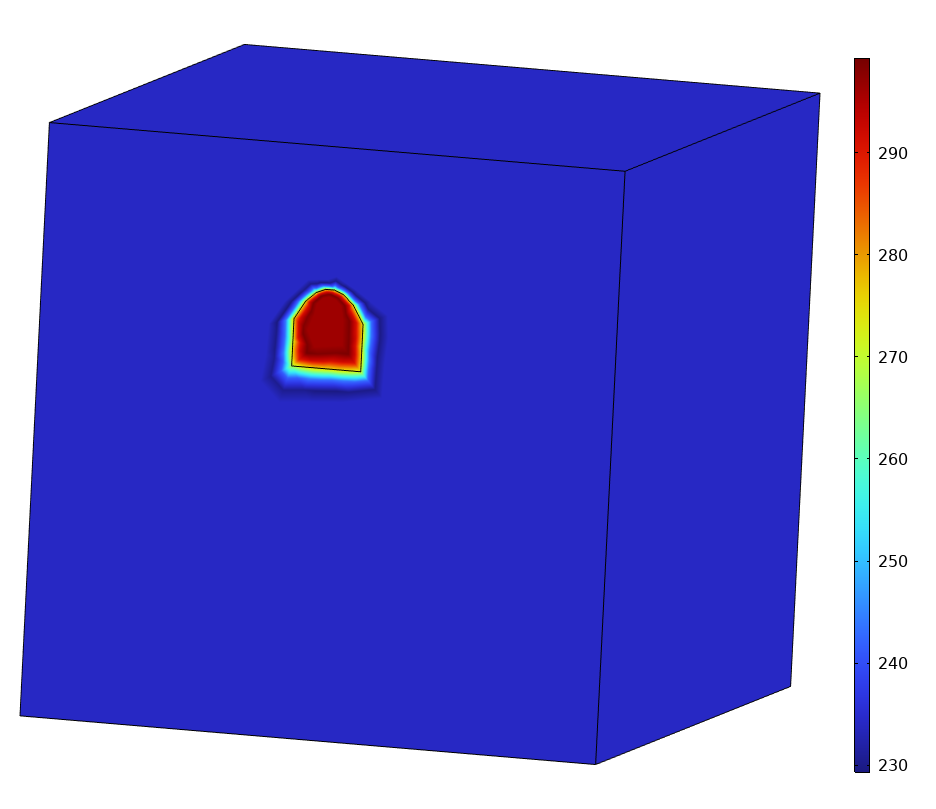 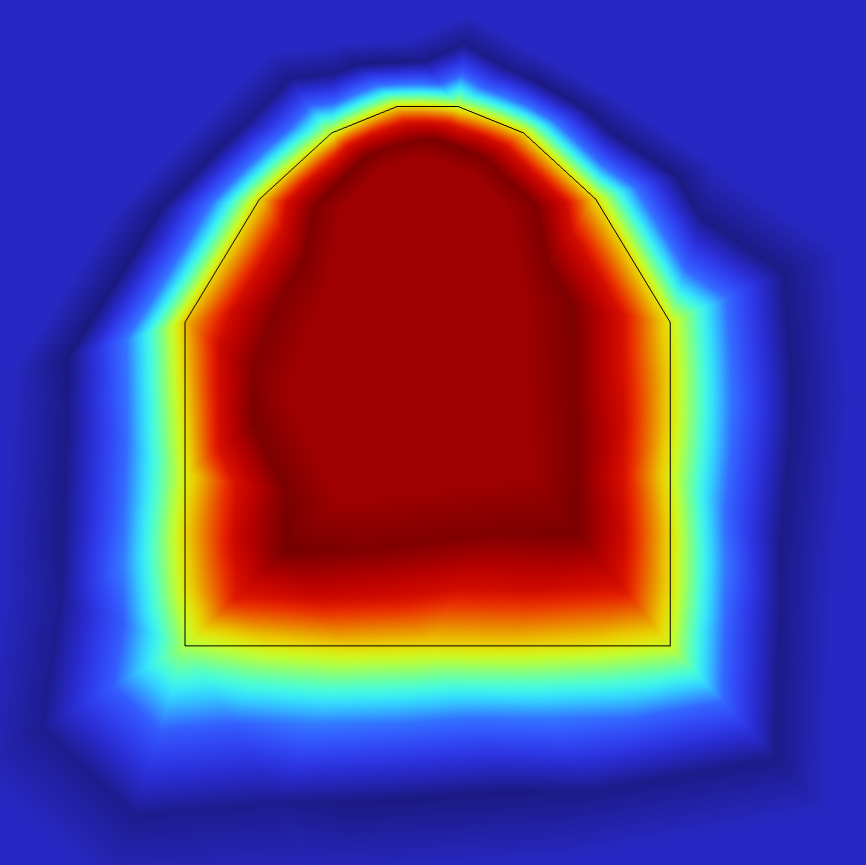 | 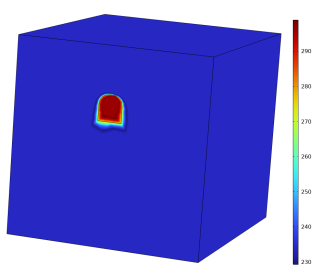 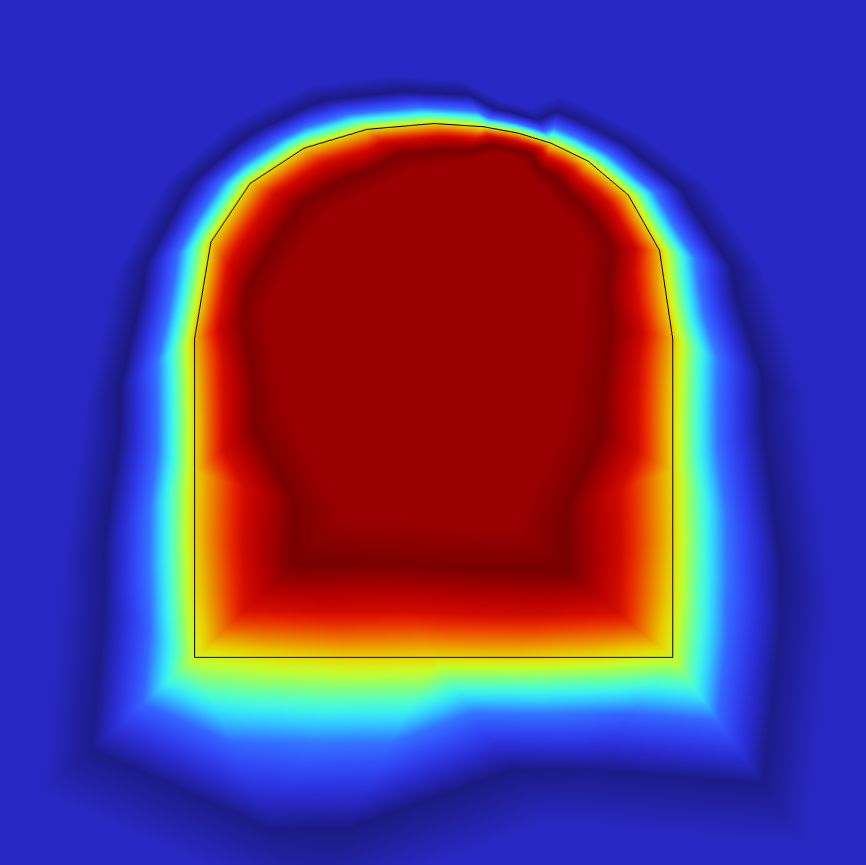 |

**Continued Table 2**

|  | **Two-centered arch (Regolith)** | **Catenary arch (Regolith)** | **Eggshell arch (Regolith)** |
| --- | --- | --- | --- |
| Martian Day (Sol) 12 | 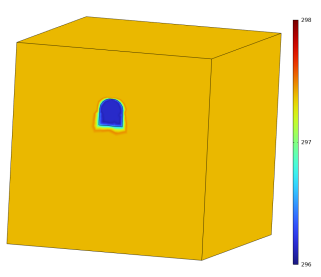 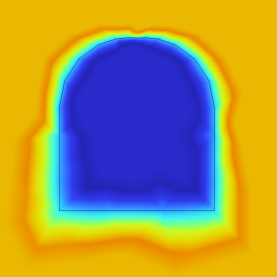 | 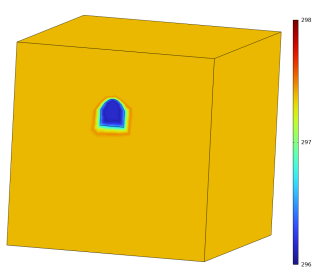 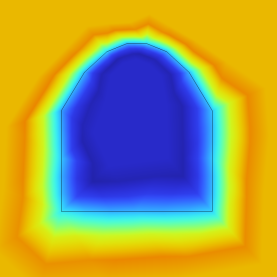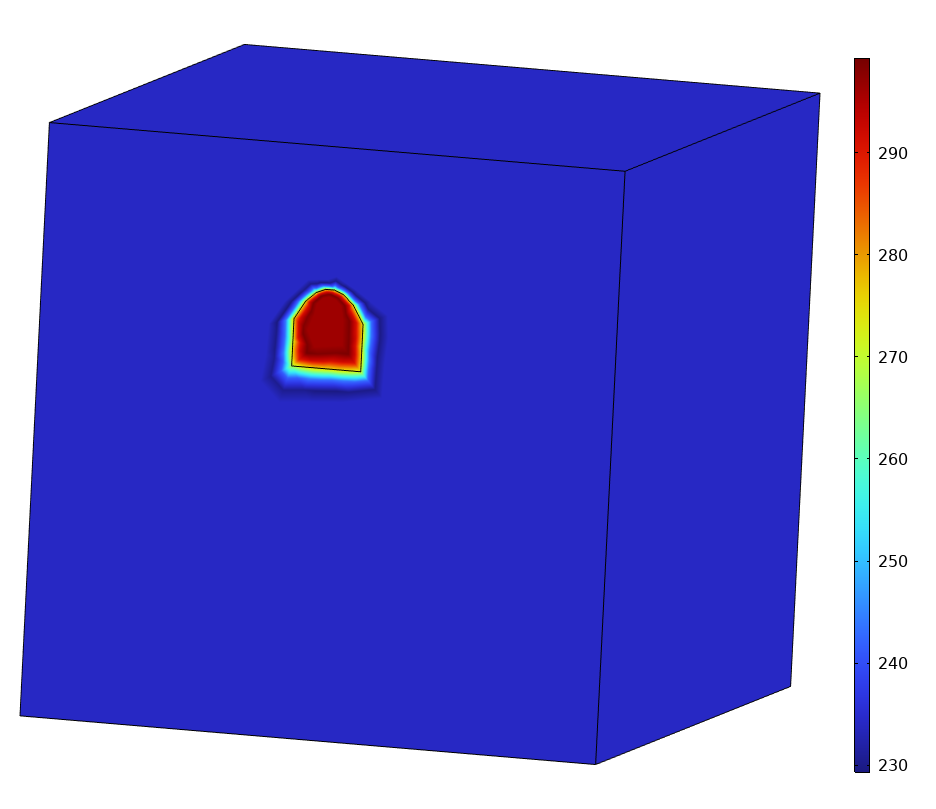 | 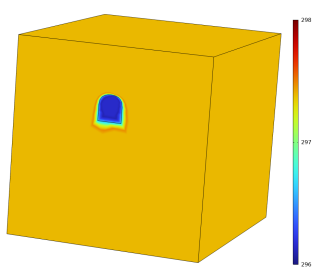 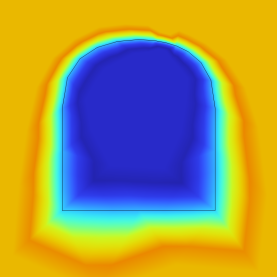 |
| Martian Day (Sol) 16 | 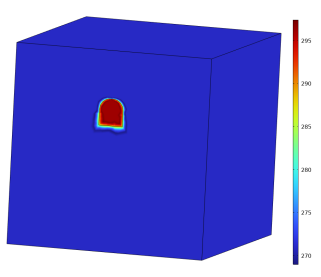 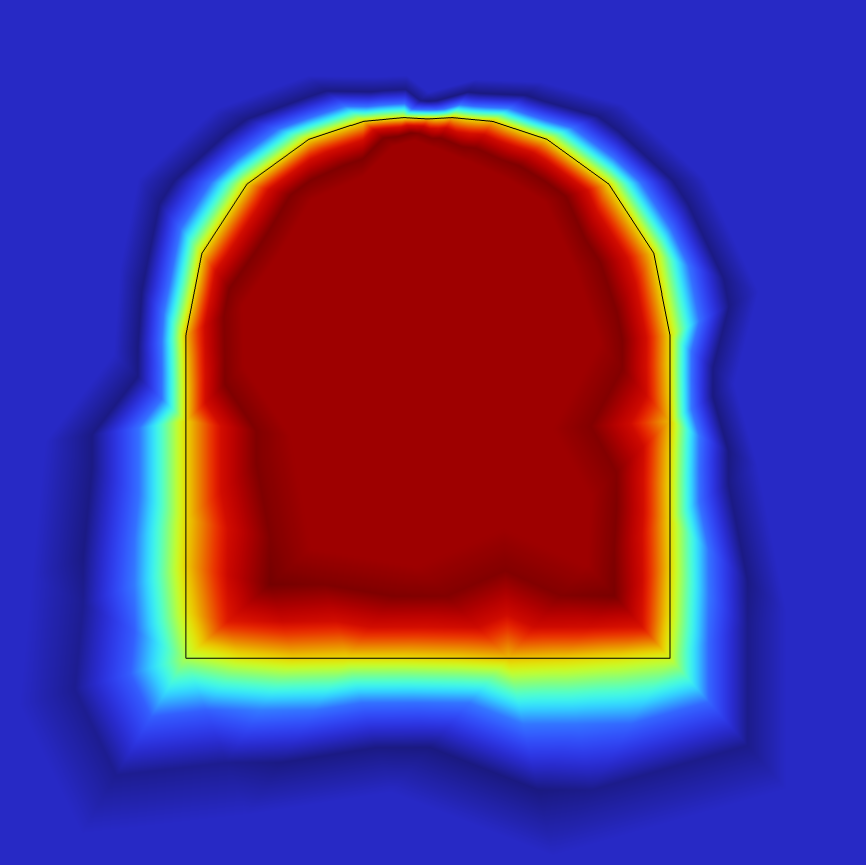 | 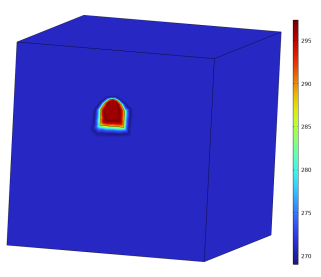 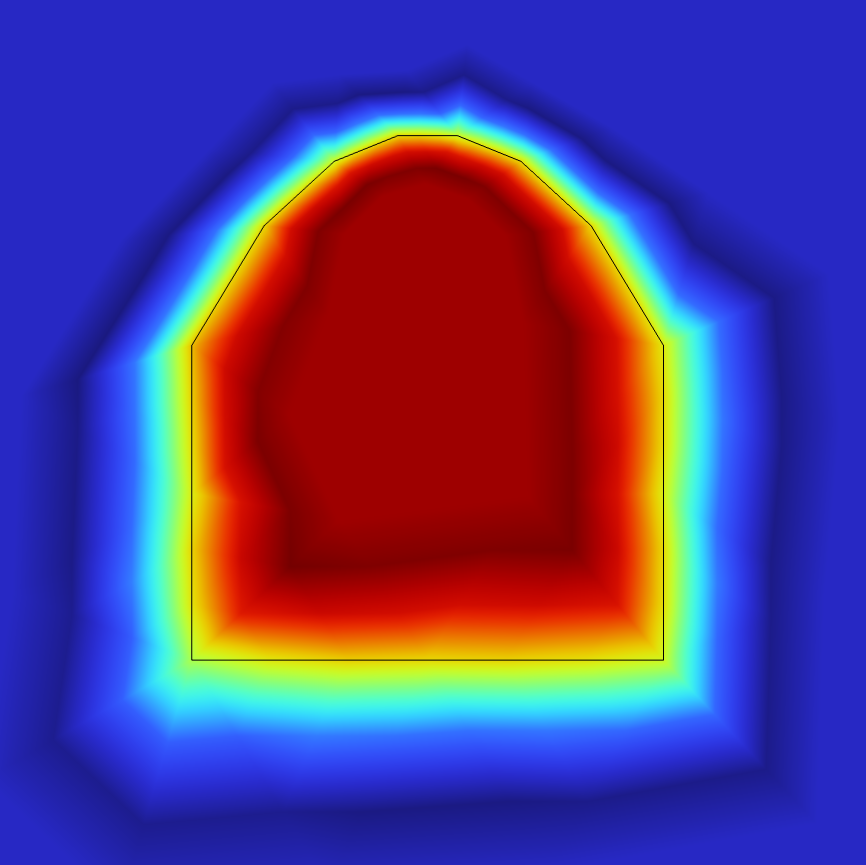 | 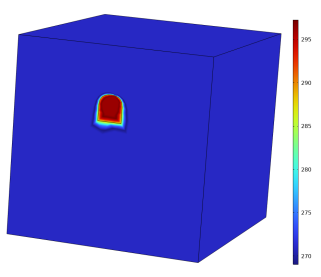 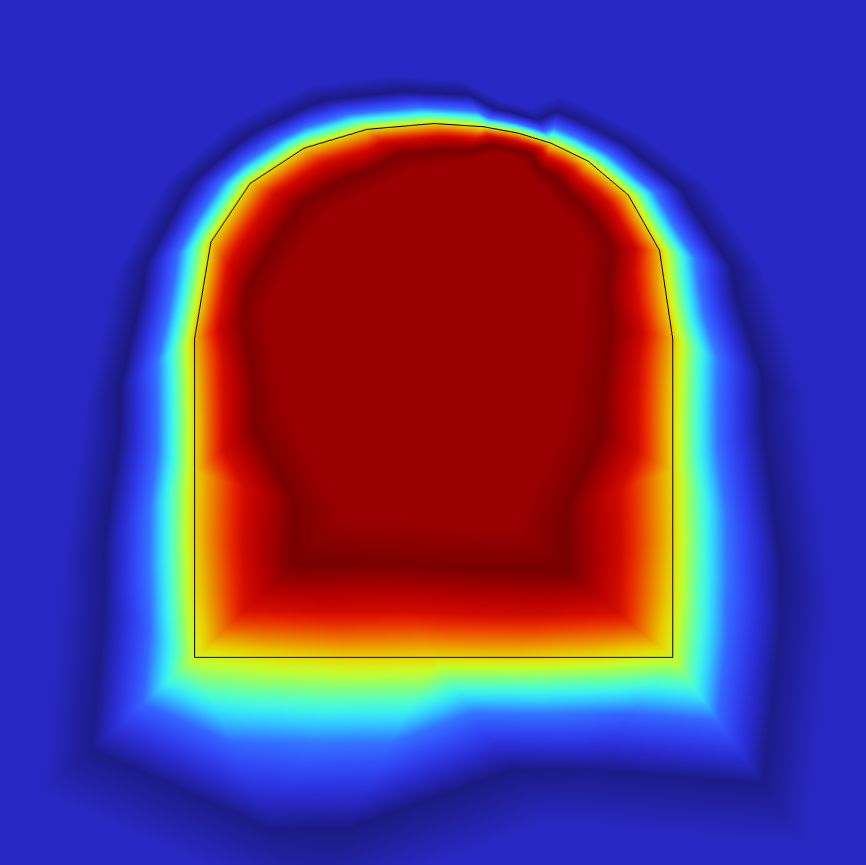 |
| Martian Day (Sol) 20 | 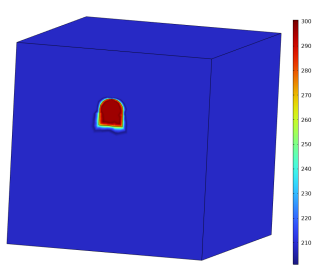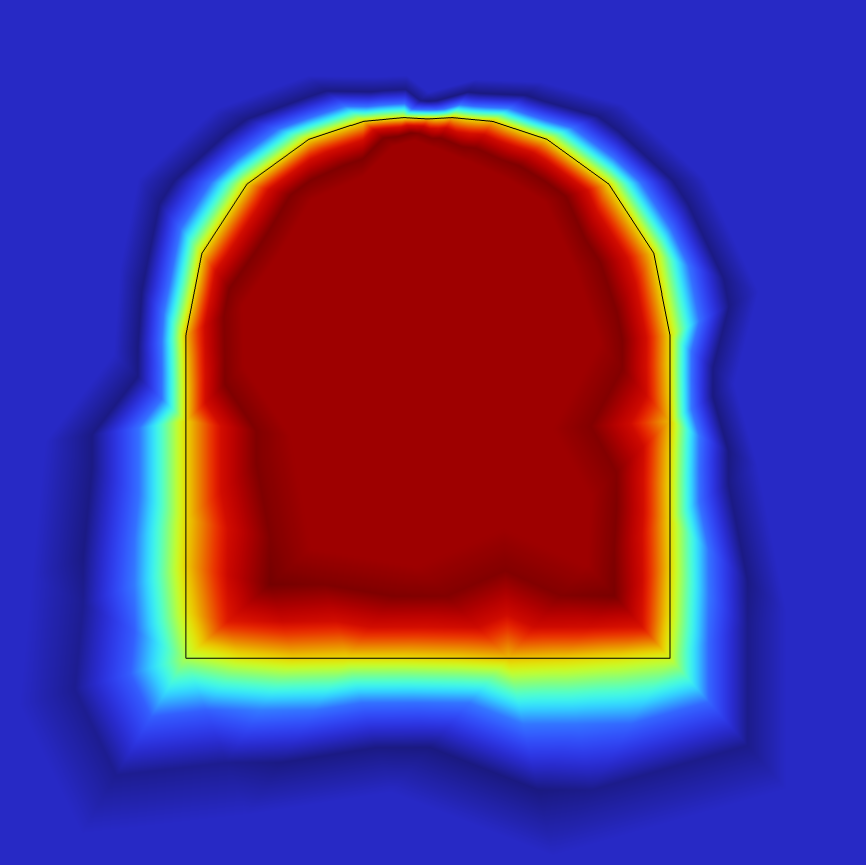 | 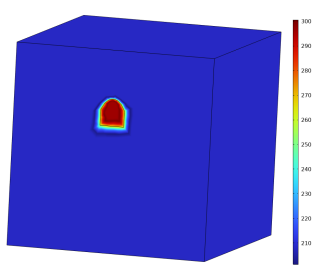 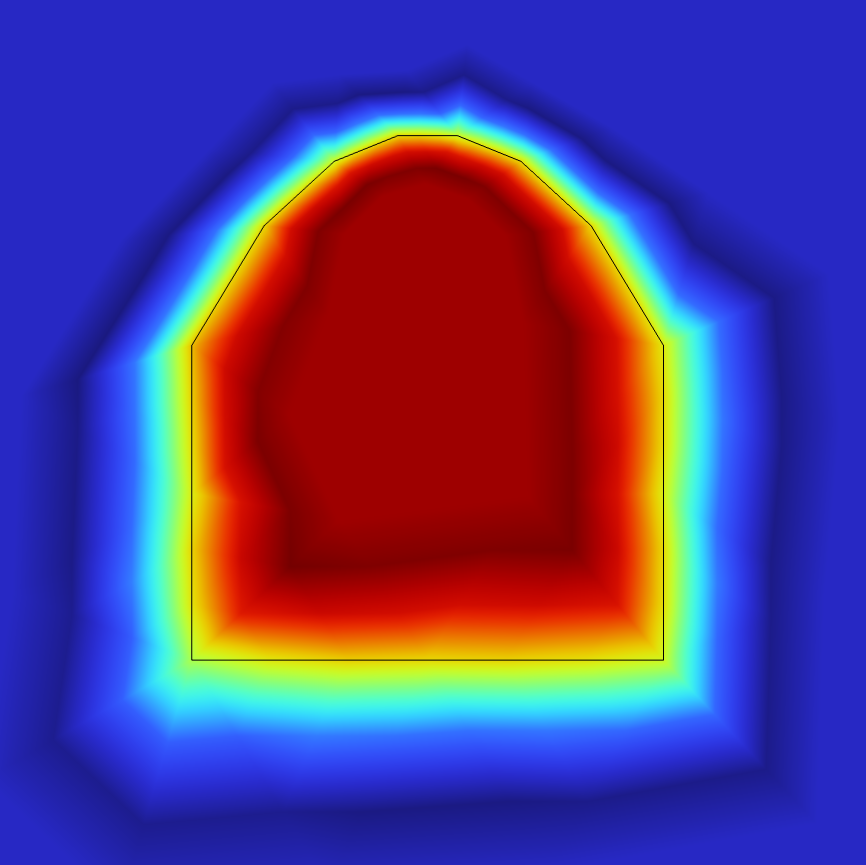 | 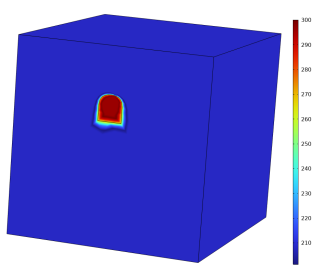 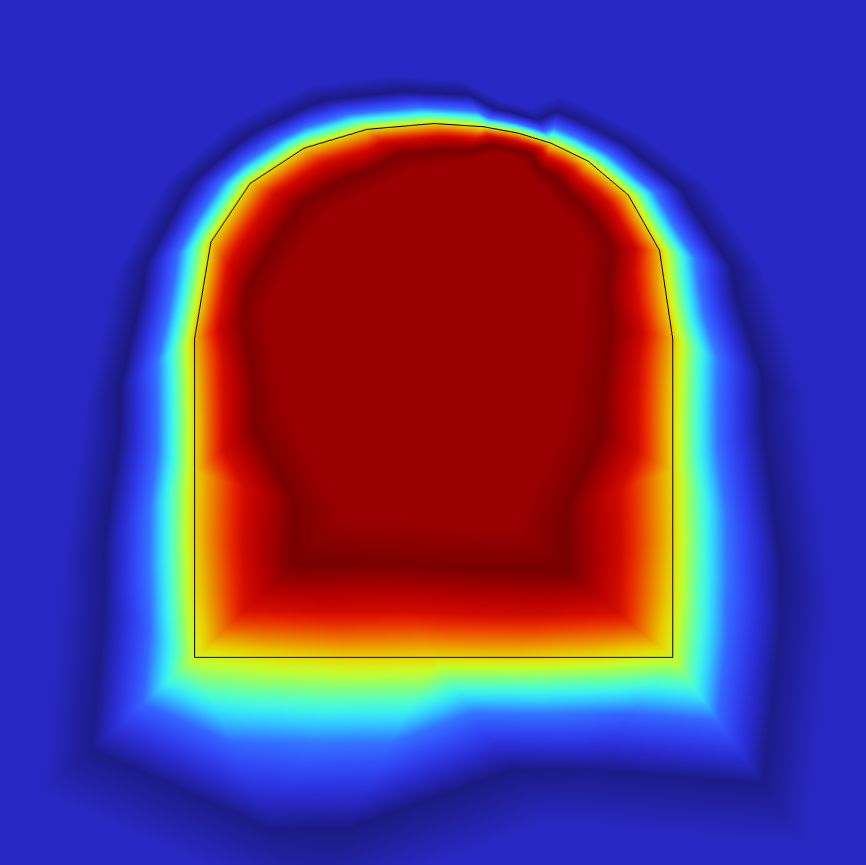 |

**Supplementary Table 3 Summary of temperature field distributions and detailed diagrams for three arches under different solar irradiance (Ls=90°)**

|  | **Two-centered arch (Regolith)** | **Catenary arch (Regolith)** | **Eggshell arch (Regolith)** |
| --- | --- | --- | --- |
| Martian Day (Sol) 0 | 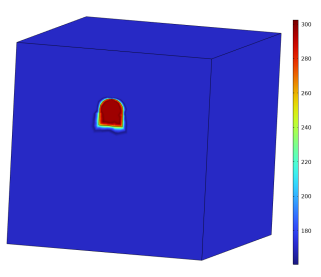 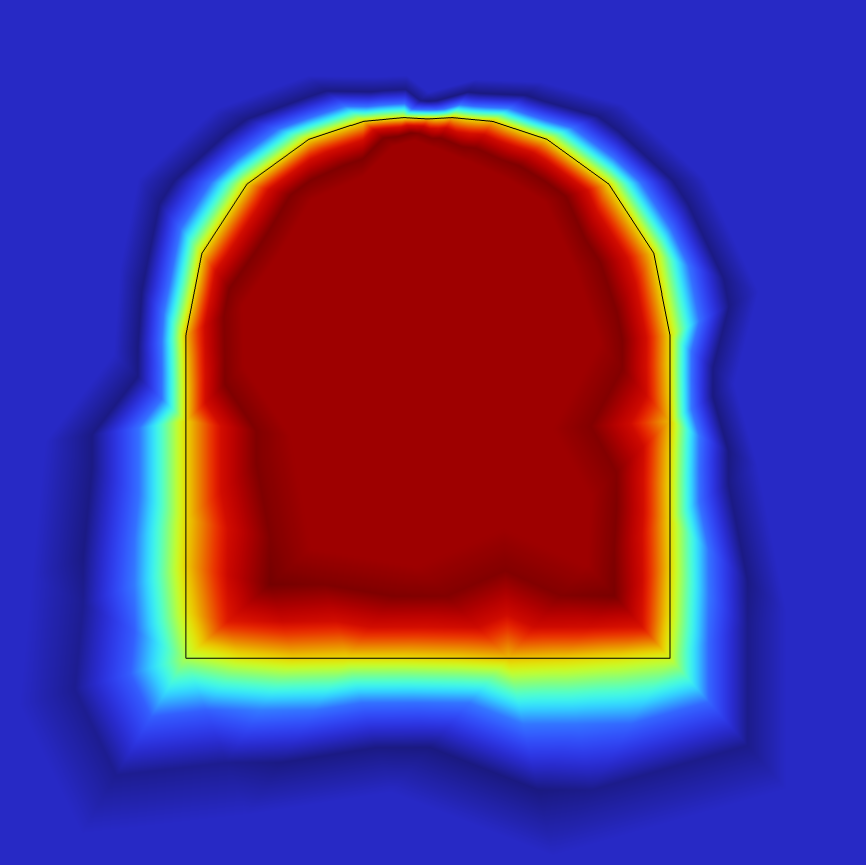 | 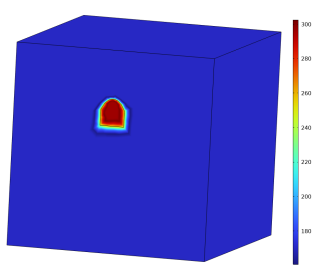 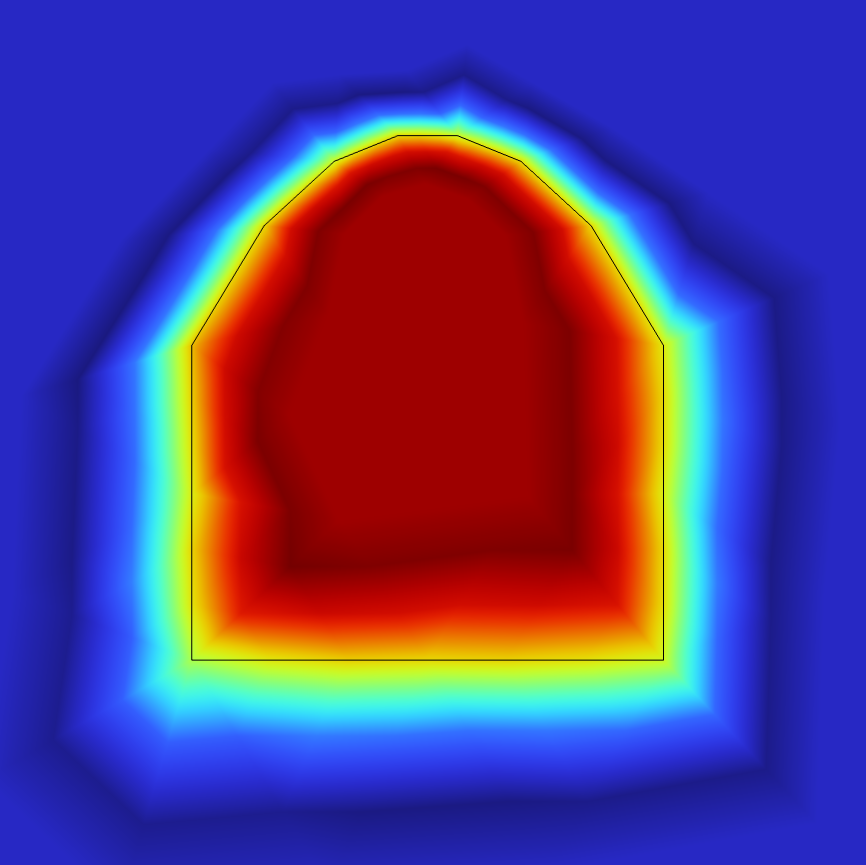 | 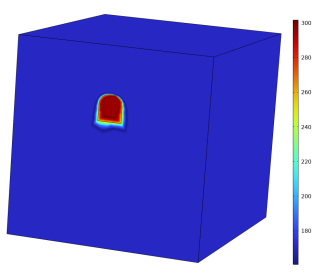 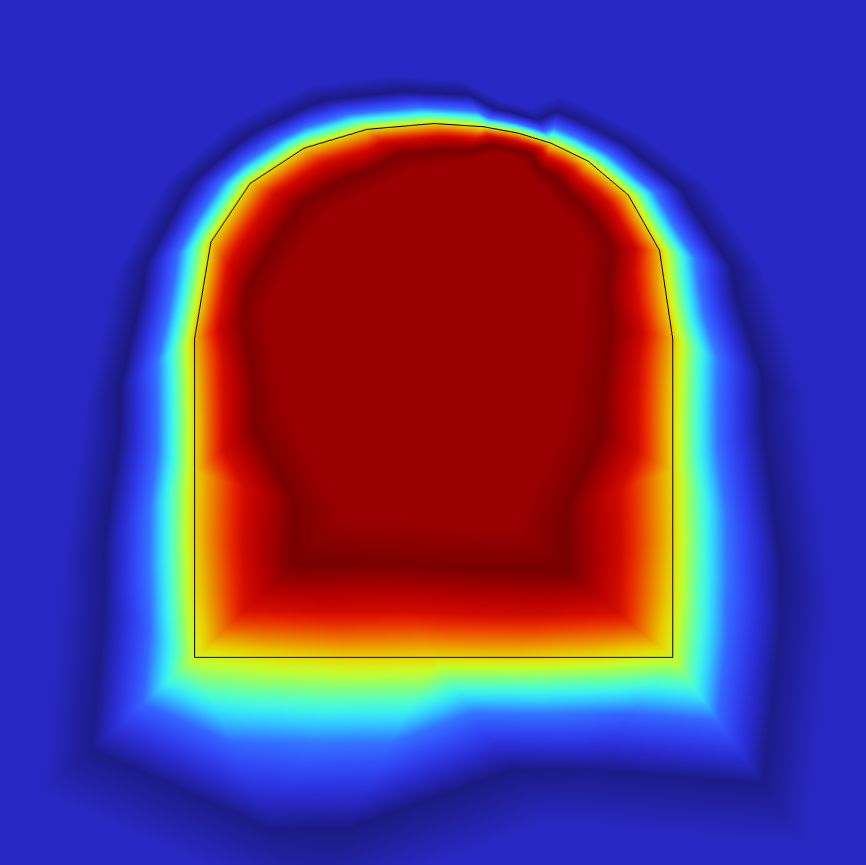 |
| Martian Day (Sol) 4 | 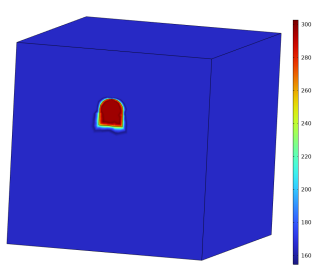 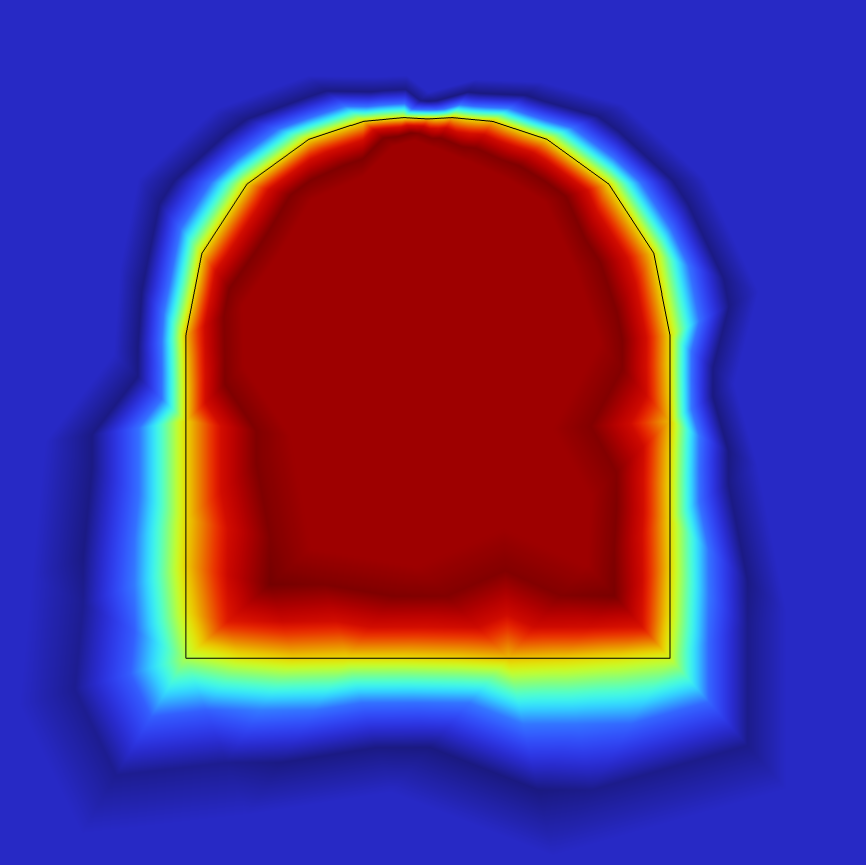 | 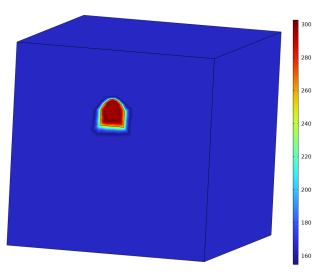 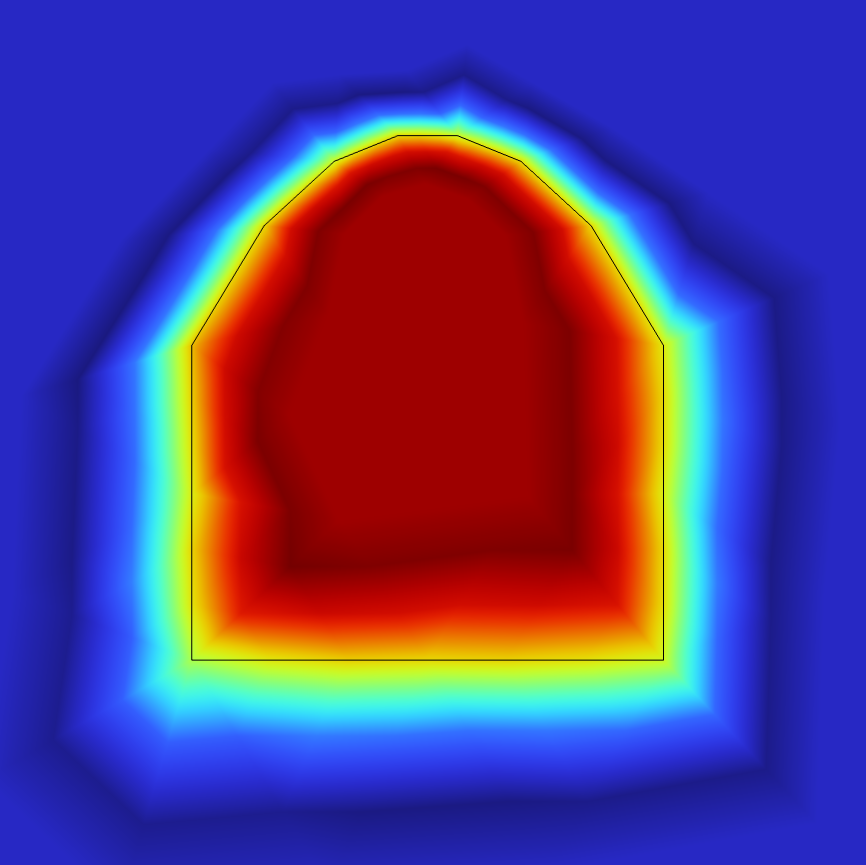 | 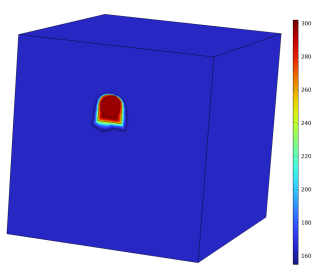 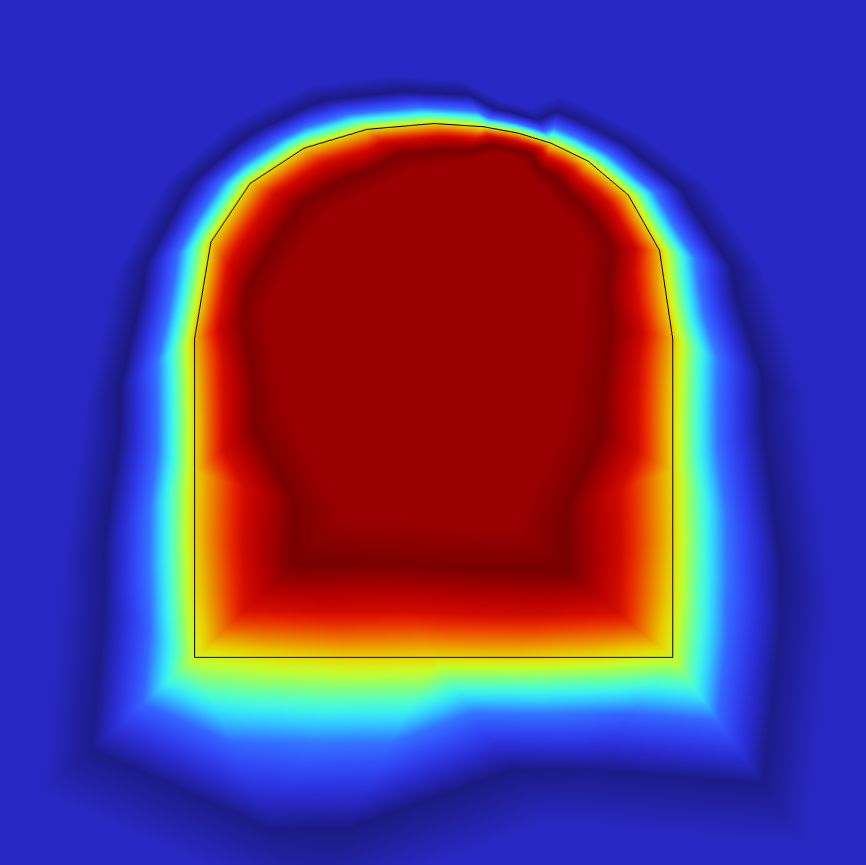 |
| Martian Day (Sol) 8 | 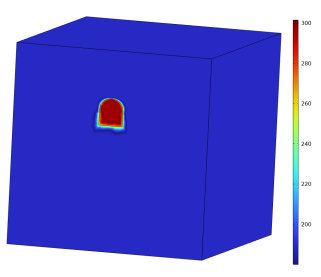 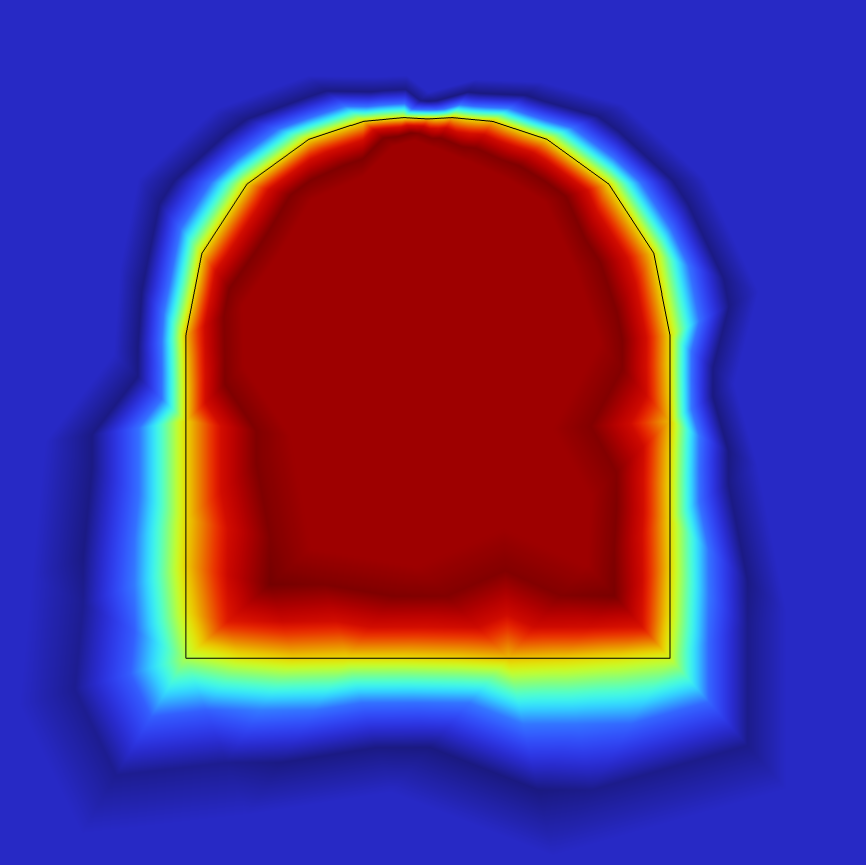 | 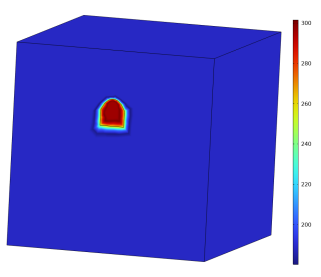 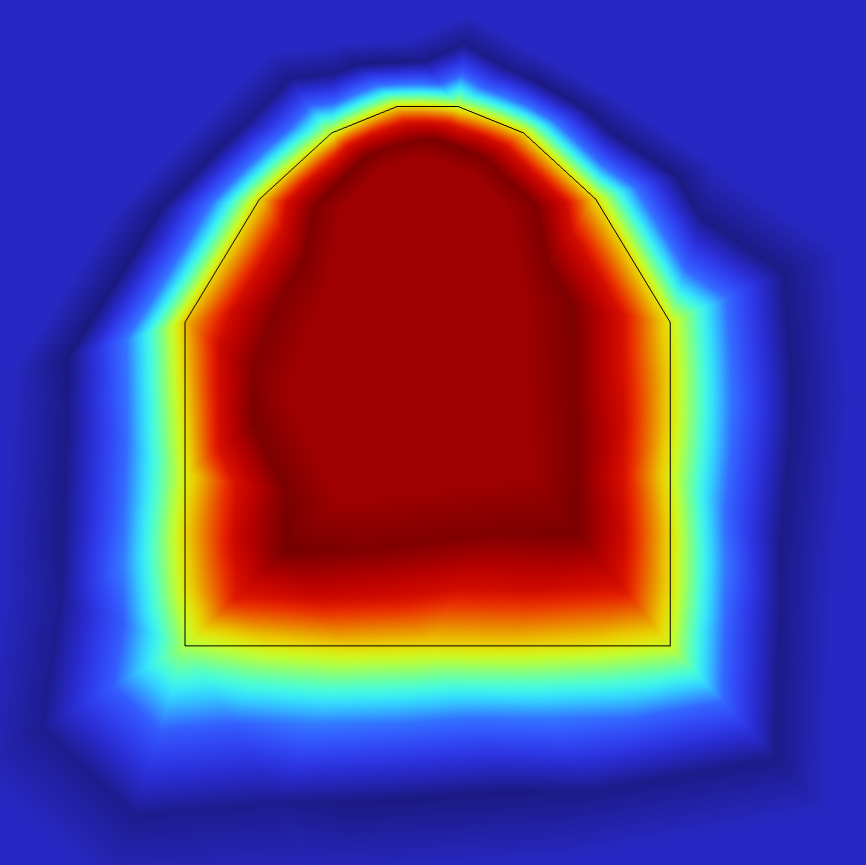 | 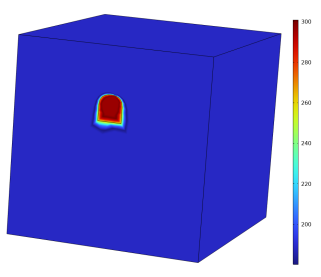 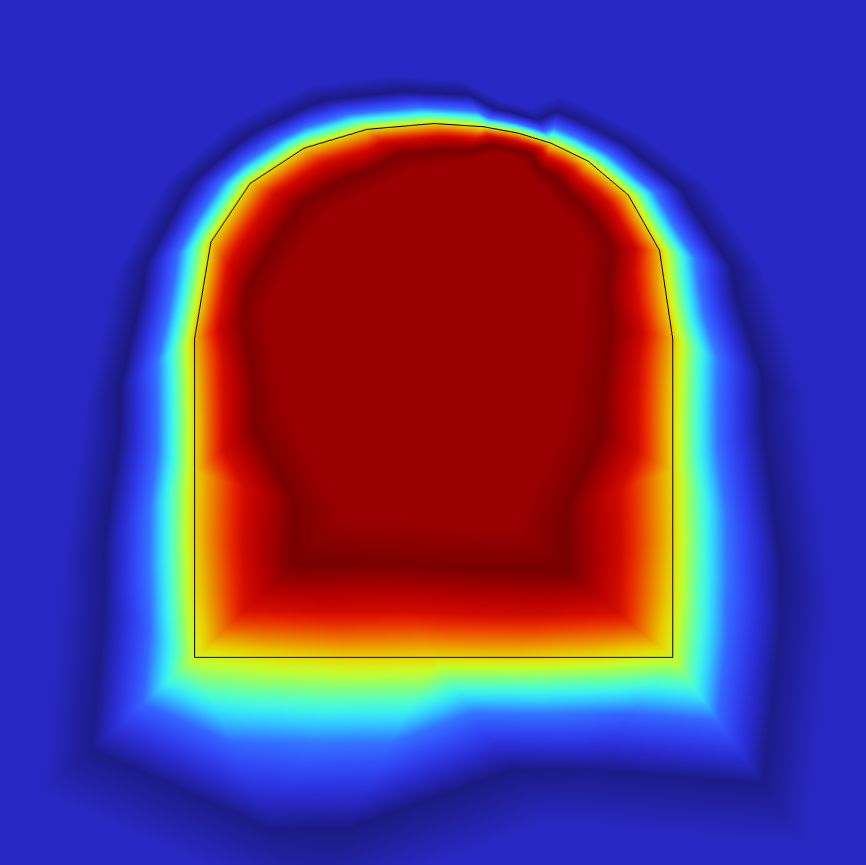 |

**Continued Table 3**

|  | **Two-centered arch (Regolith)** | **Catenary arch (Regolith)** | **Eggshell arch (Regolith)** |
| --- | --- | --- | --- |
| Martian Day (Sol) 12 | 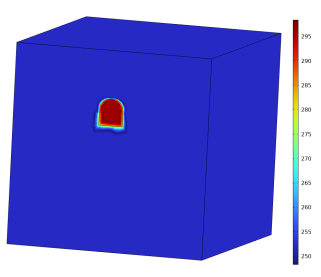 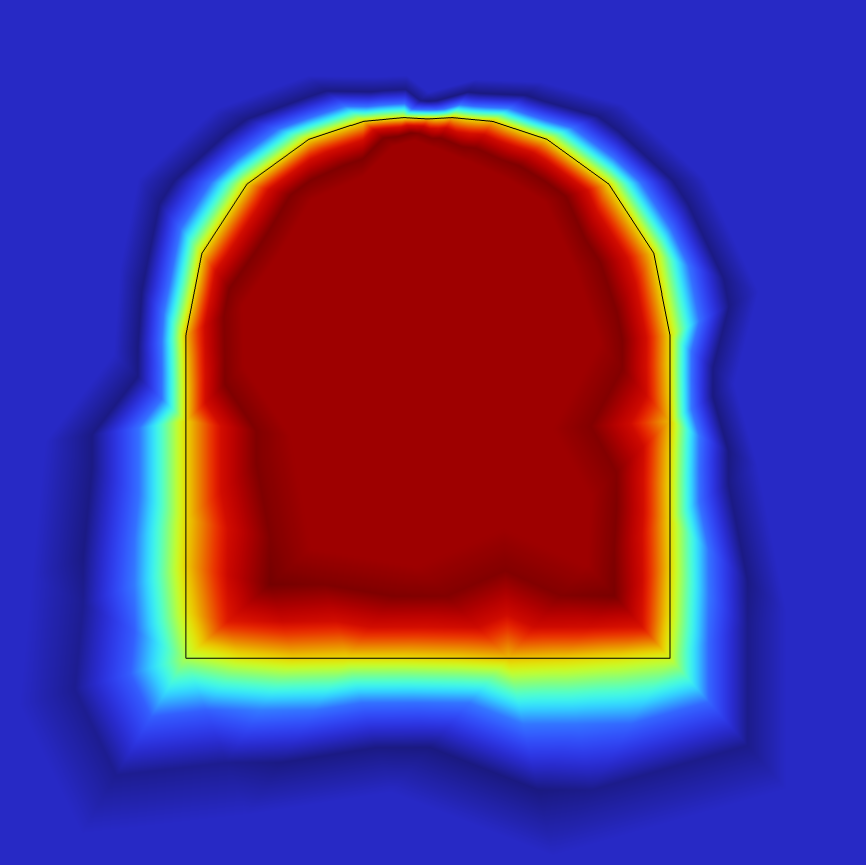 | 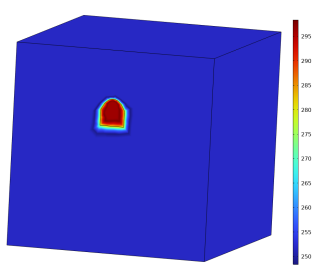 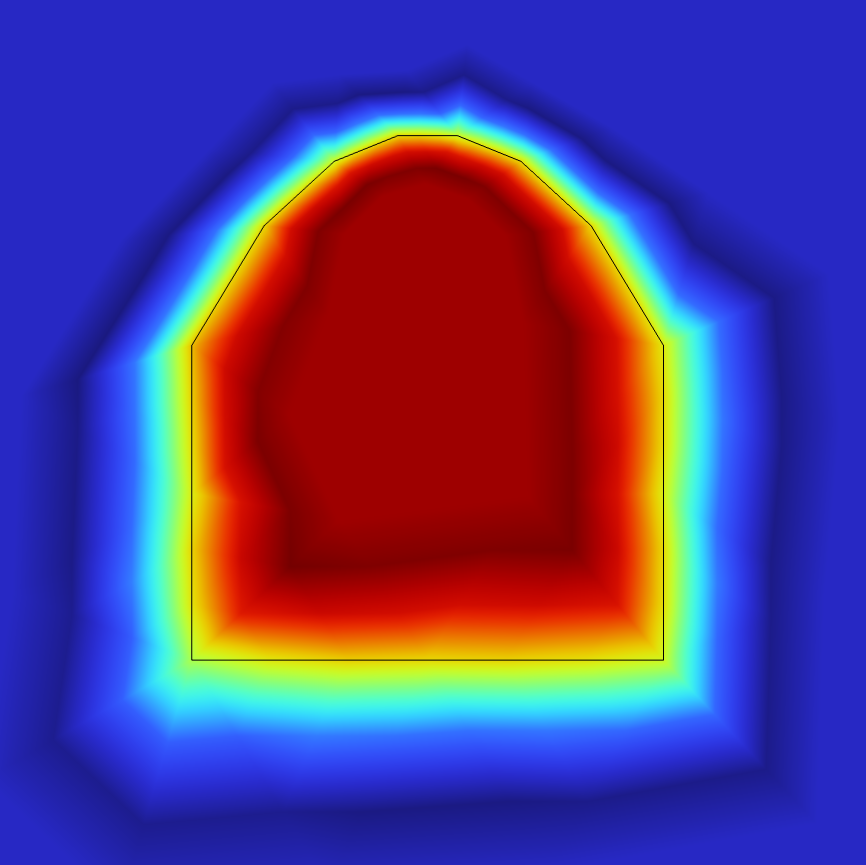 | 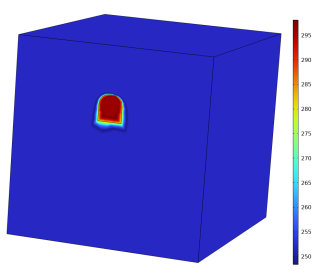 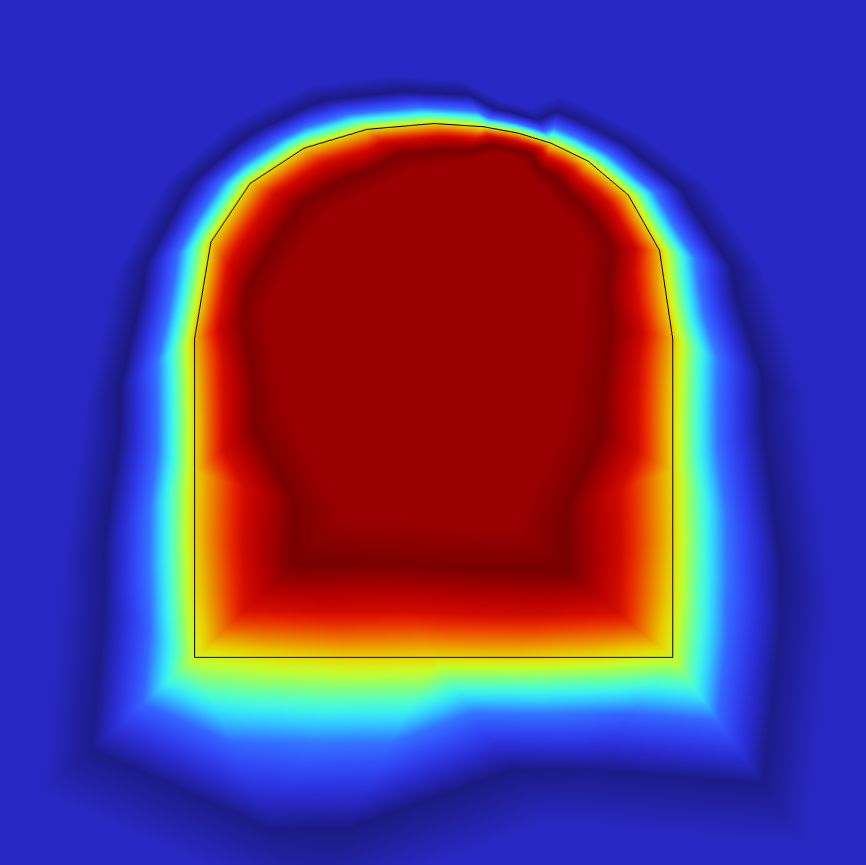 |
| Martian Day (Sol) 16 | 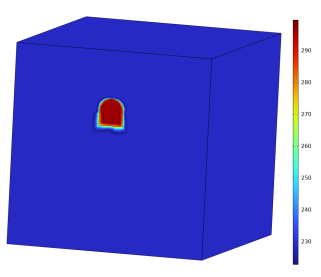 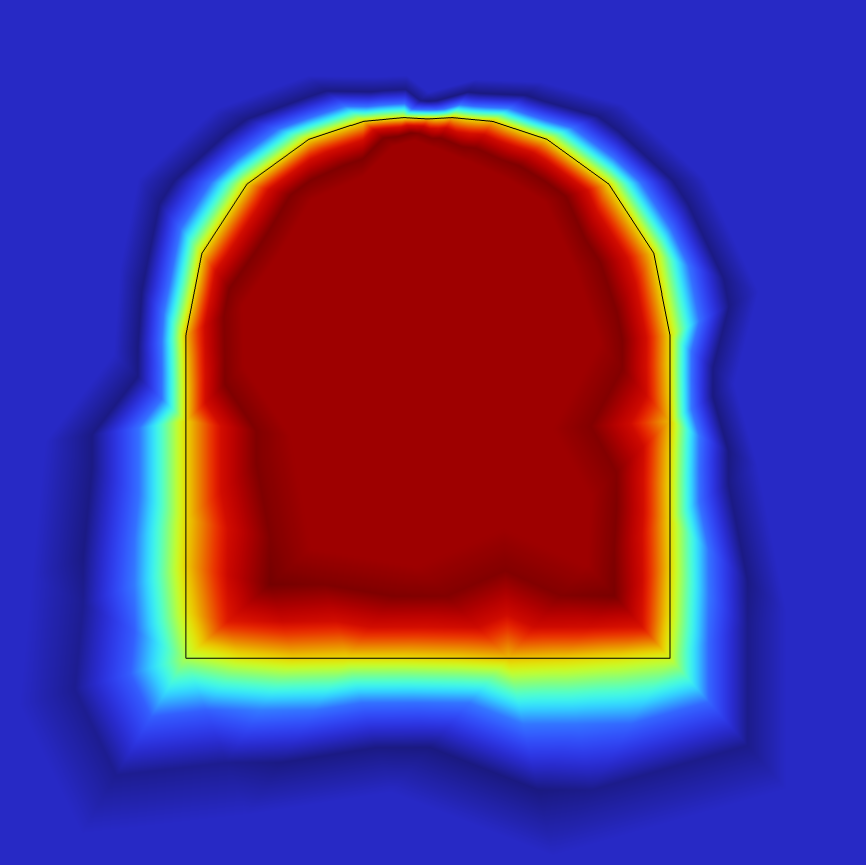 | 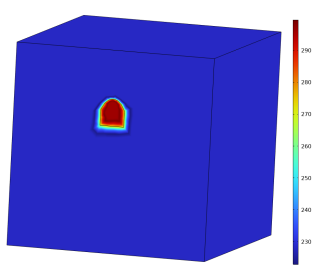 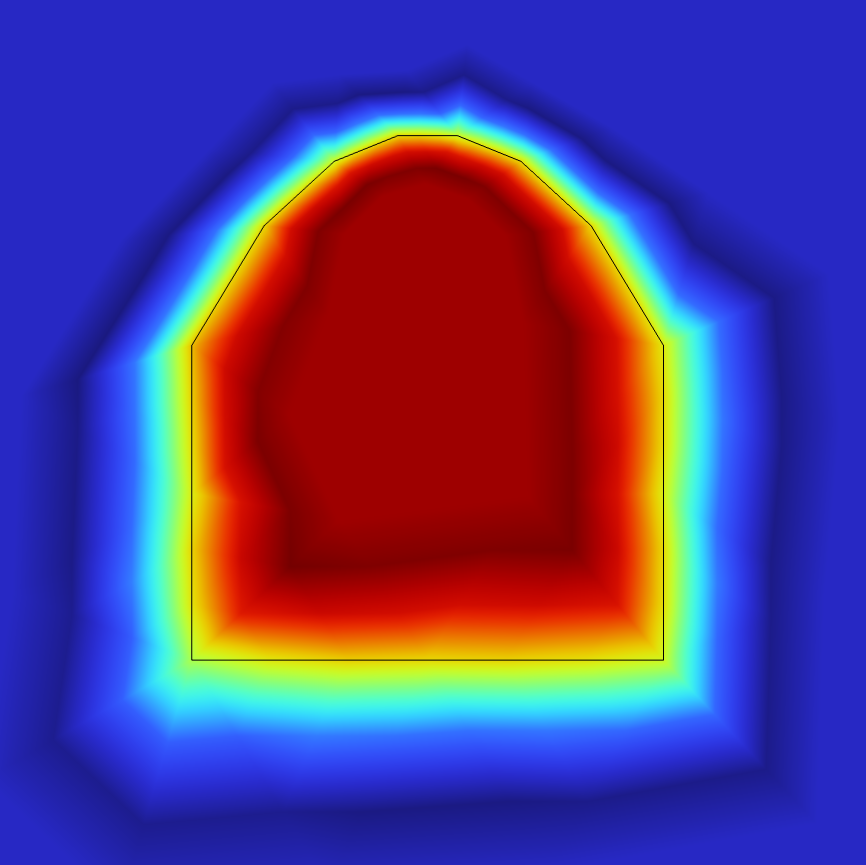 | 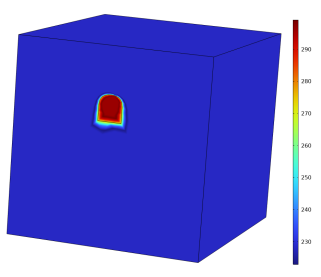 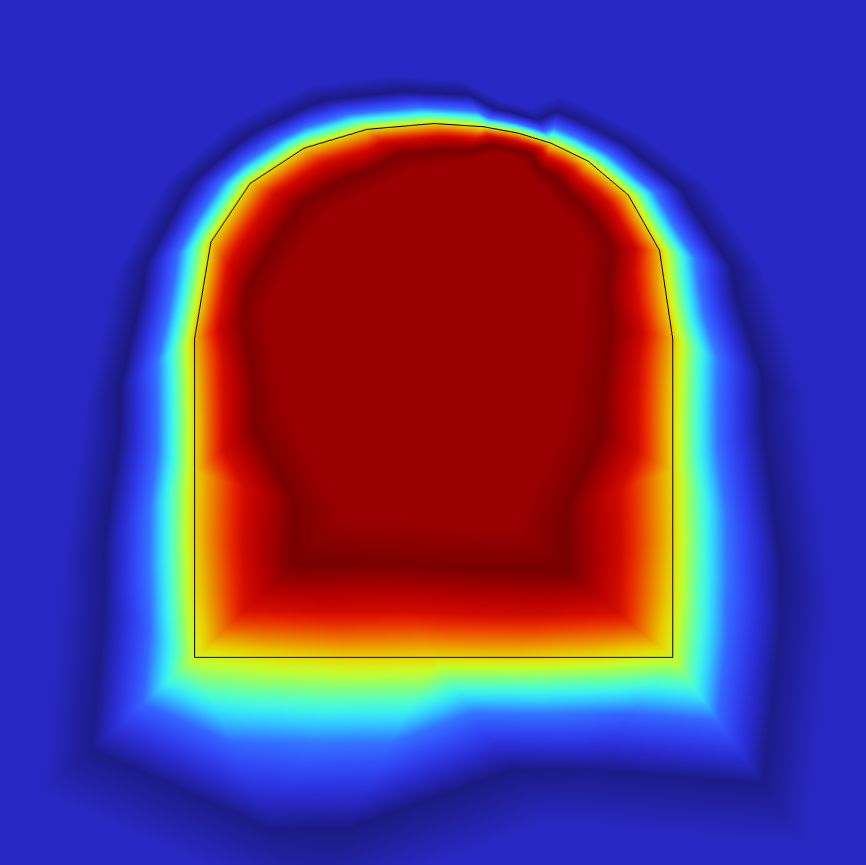 |
| Martian Day (Sol) 20 | 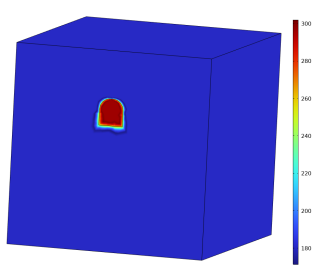 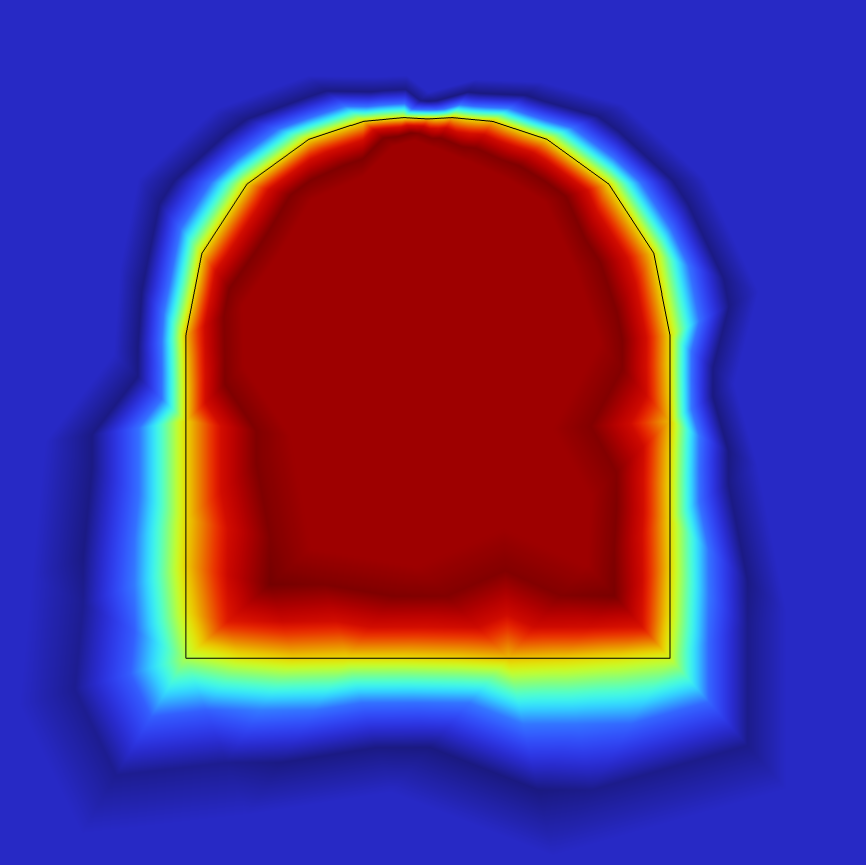 | 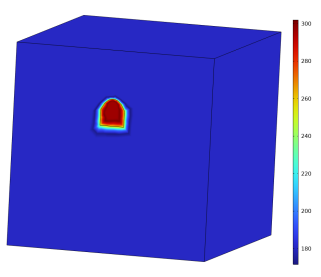 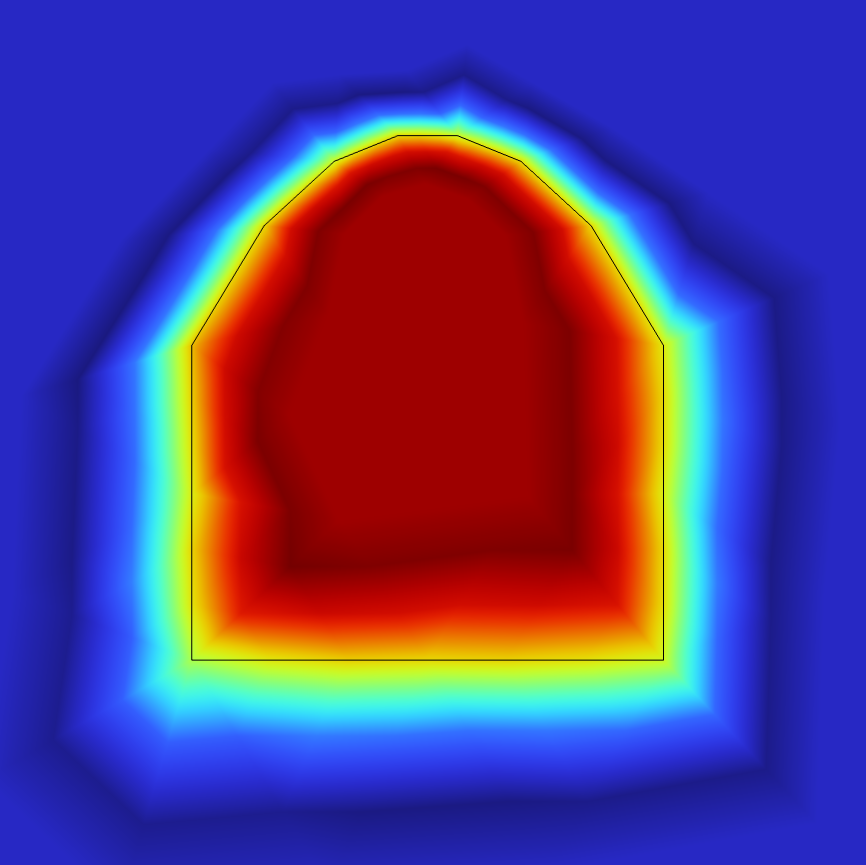 | 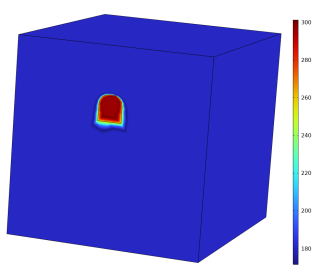 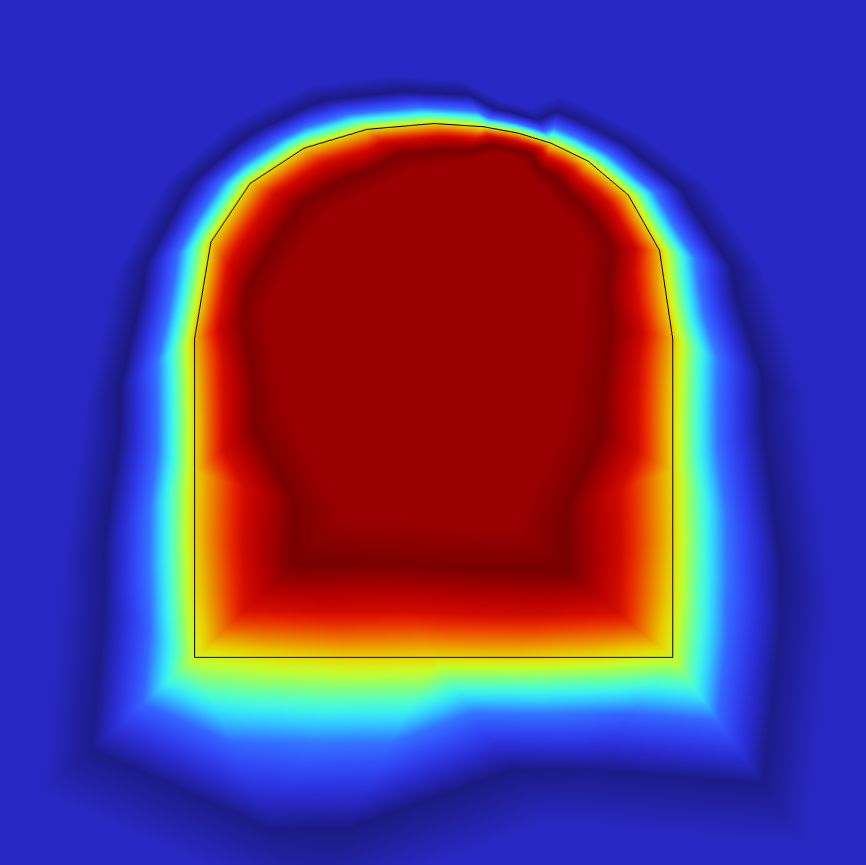 |

**Supplementary Table 4 Summary of temperature field distributions and detailed diagrams for three arches under different solar irradiance (General)**

|  | **Two-centered arch (Regolith)** | **Catenary arch (Regolith)** | **Eggshell arch (Regolith)** |
| --- | --- | --- | --- |
| Martian Day (Sol) 0 | 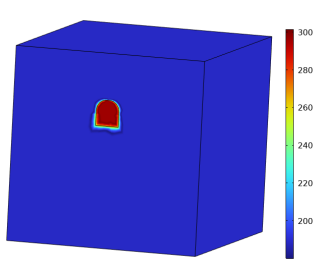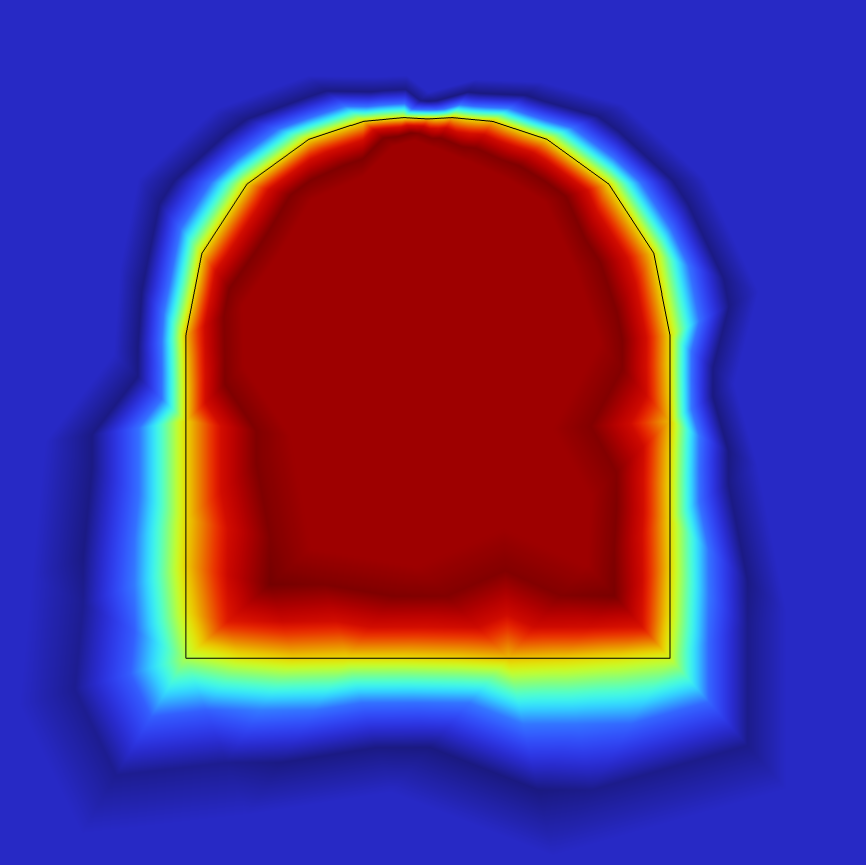 | 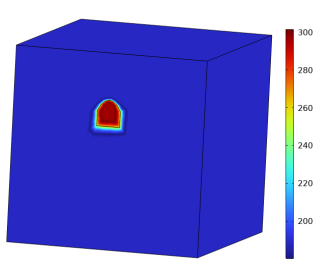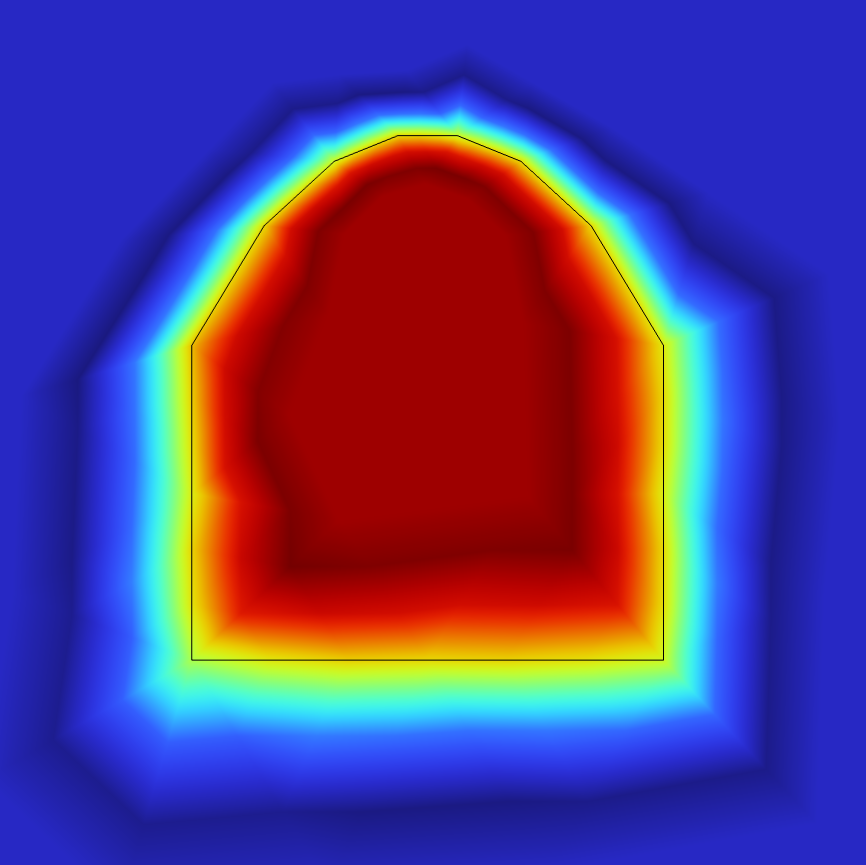 | 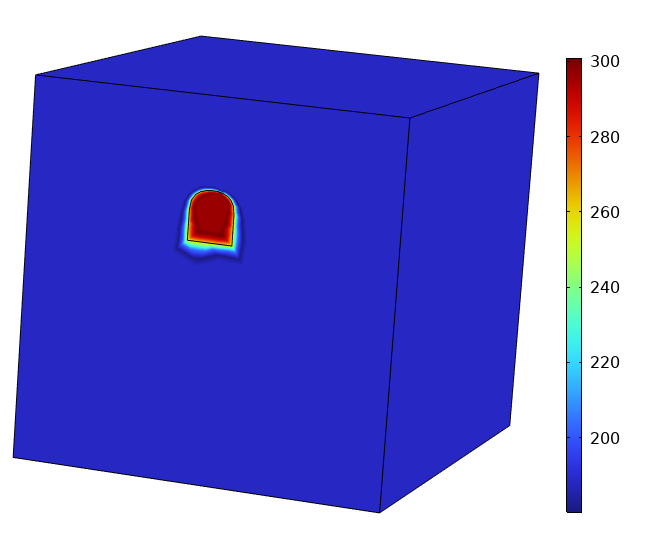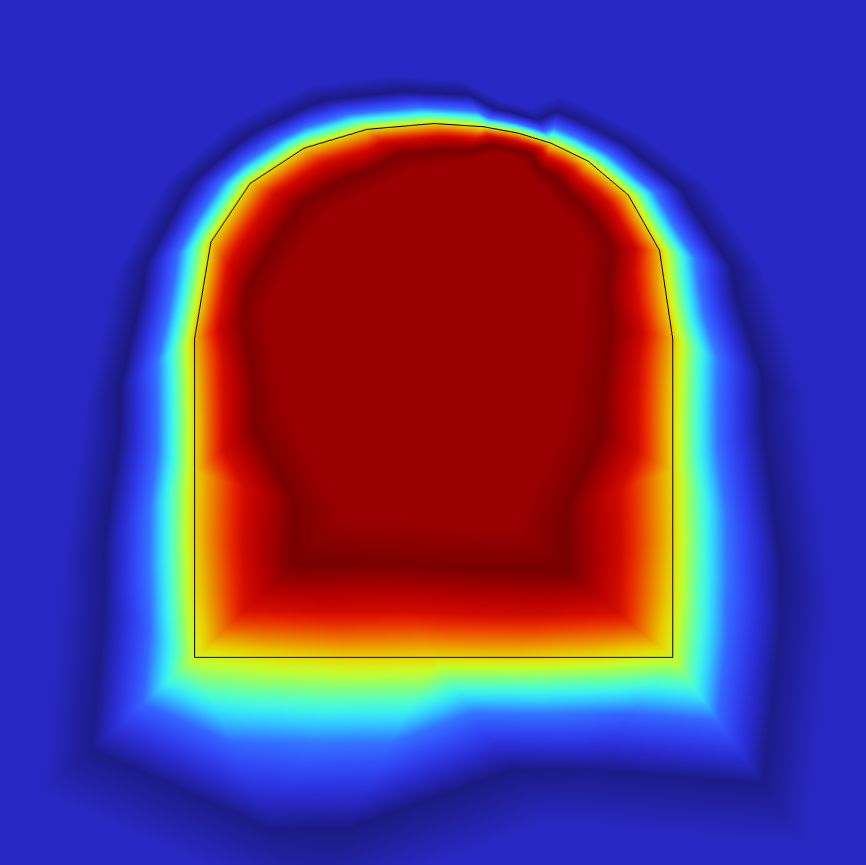 |
| Martian Day (Sol) 4 | 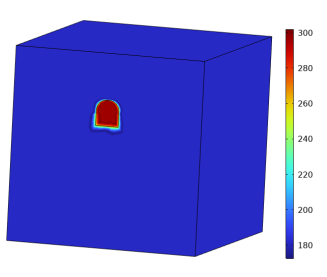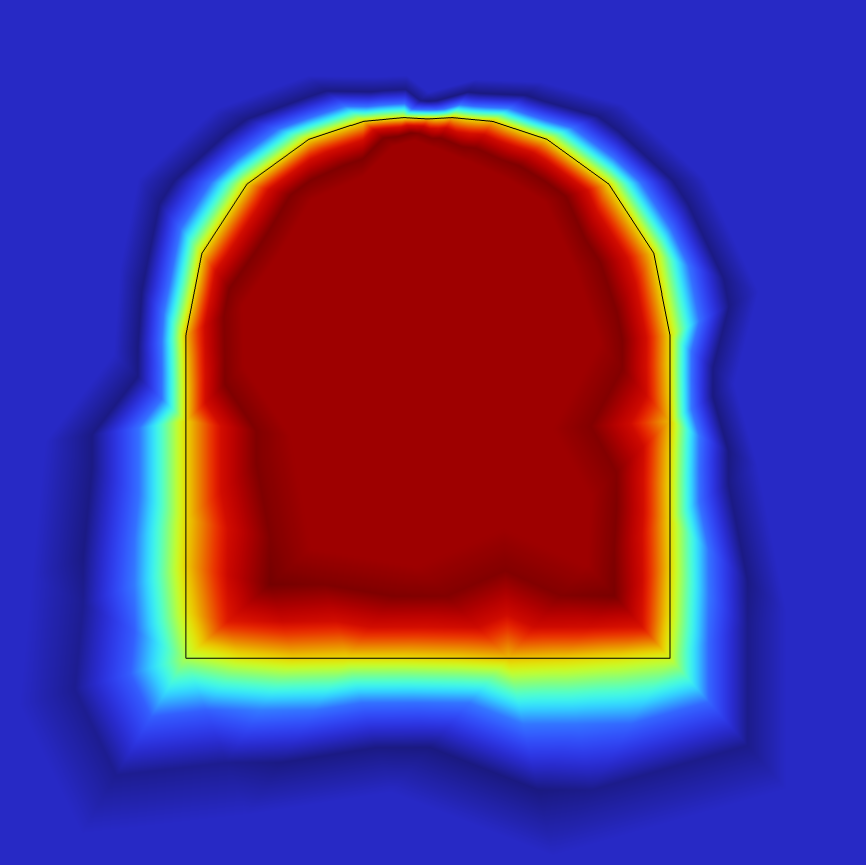 | 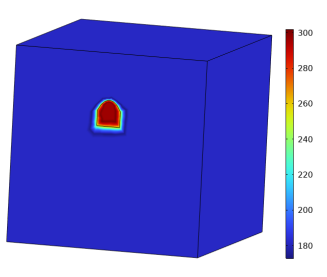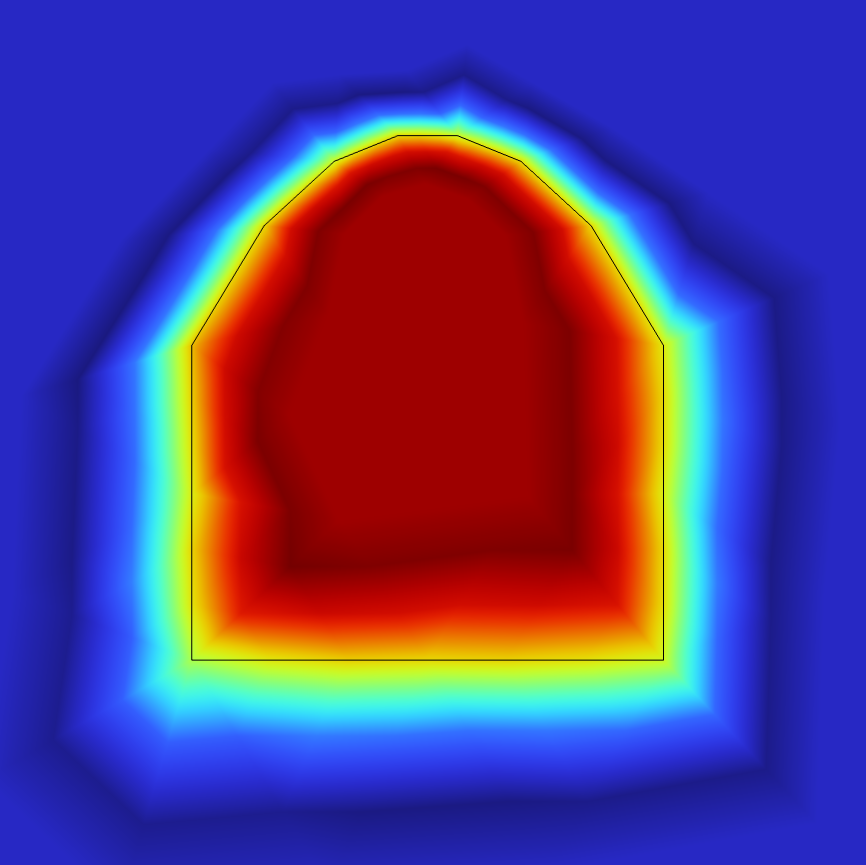 | 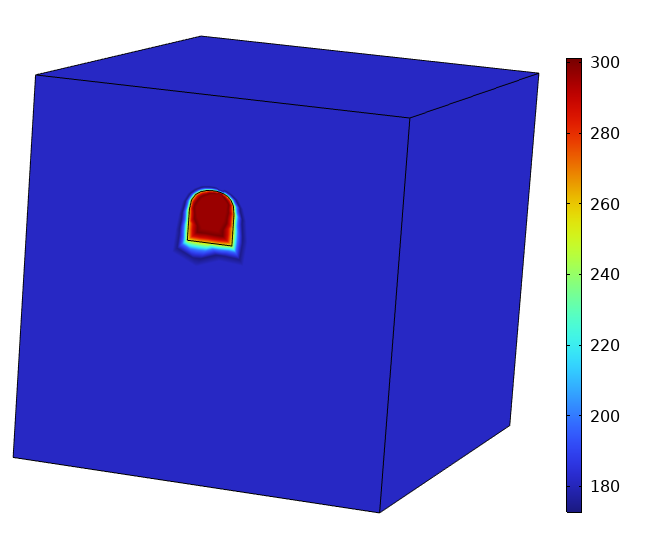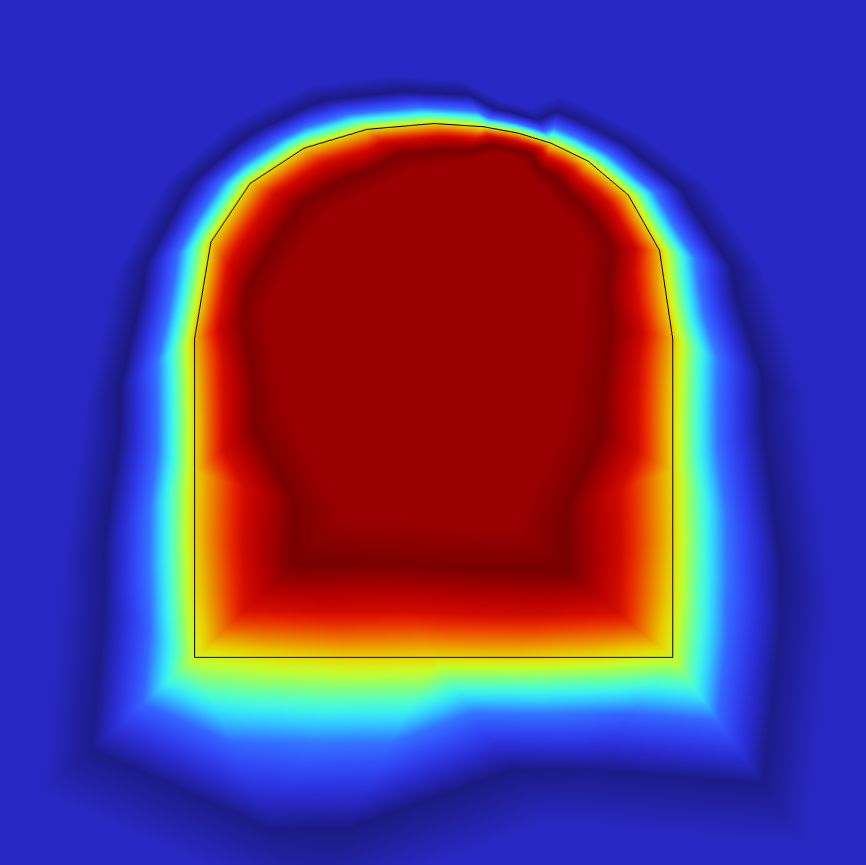 |
| Martian Day (Sol) 8 | 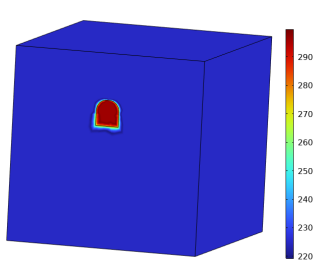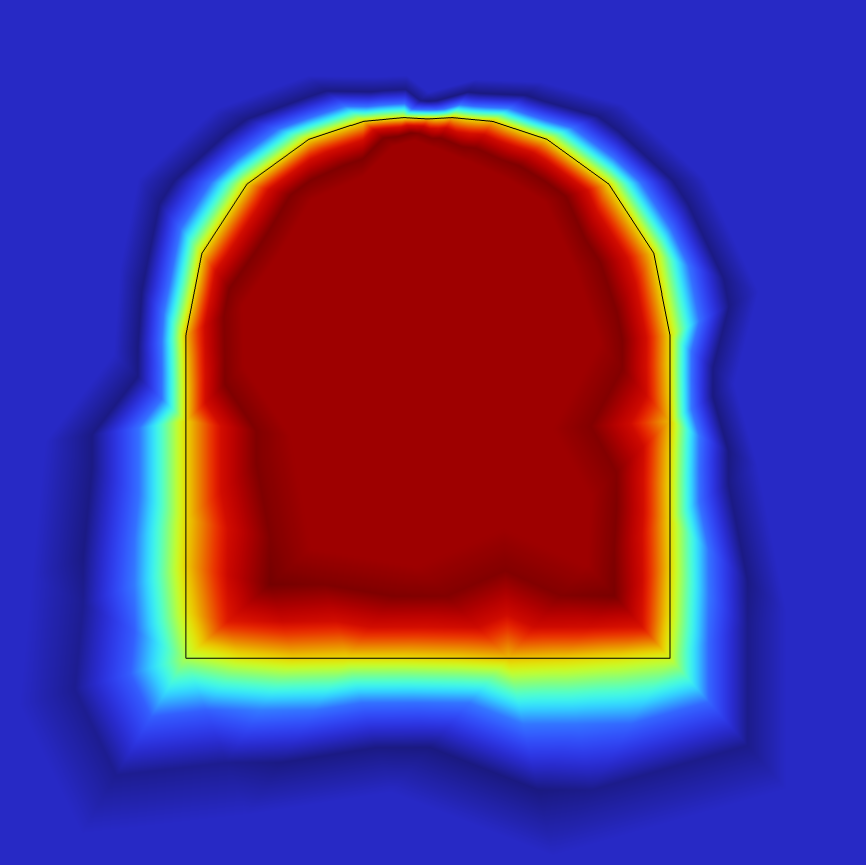 | 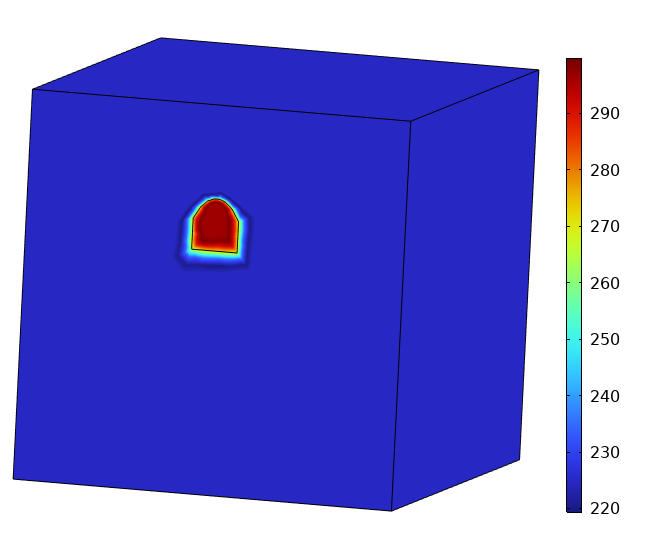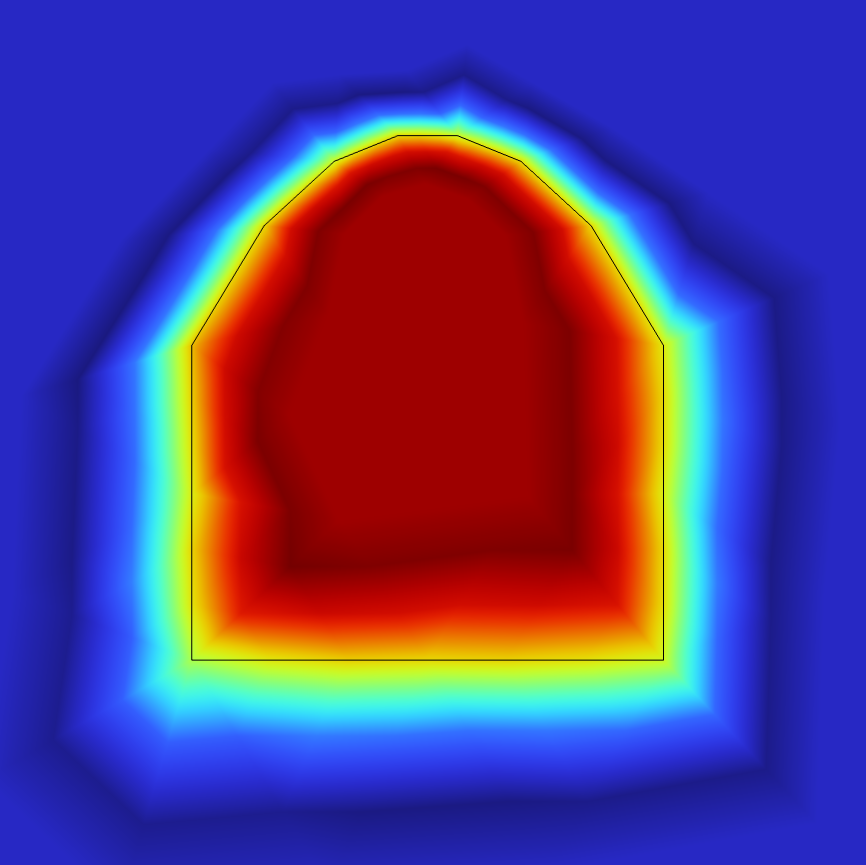 | 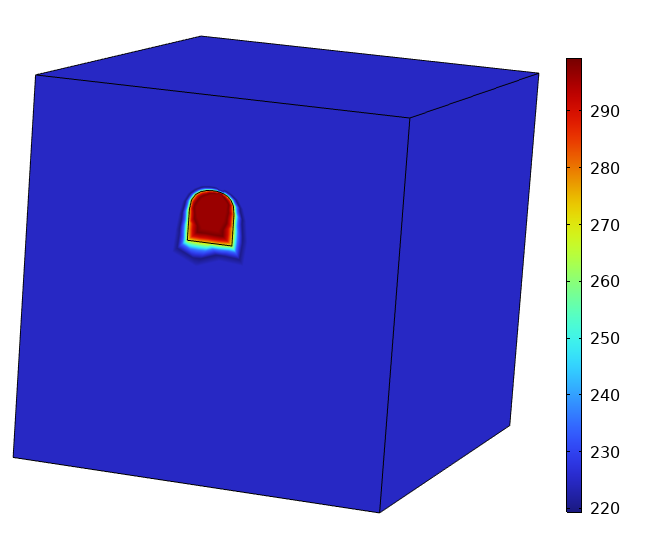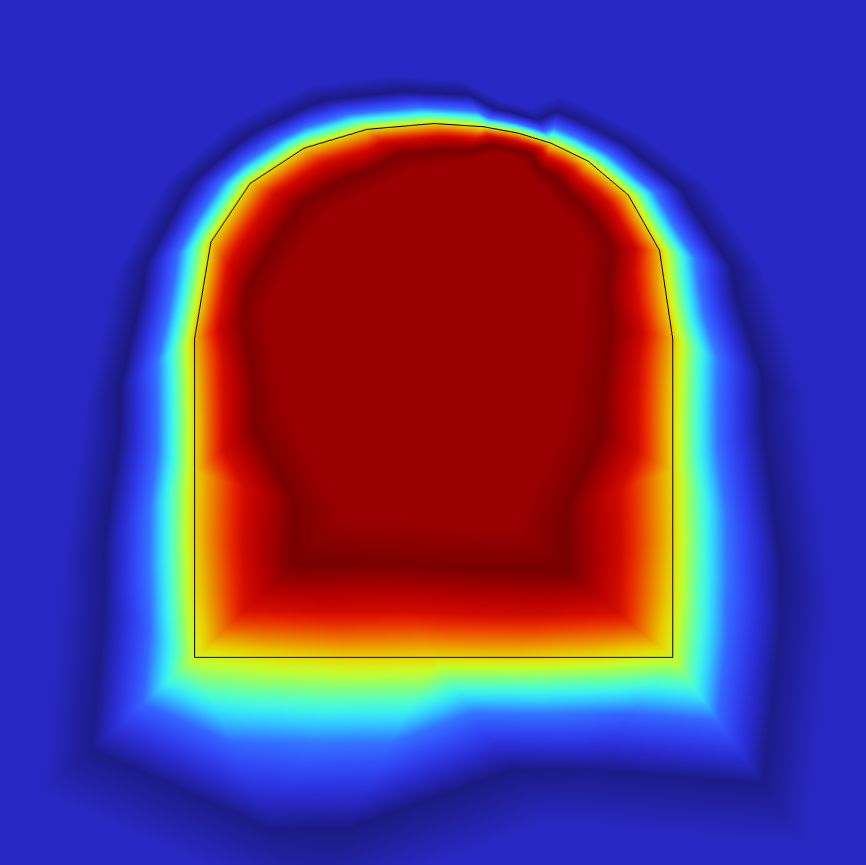 |

**Continued Table 4**

|  | **Two-centered arch (Regolith)** | **Catenary arch (Regolith)** | **Eggshell arch (Regolith)** |
| --- | --- | --- | --- |
| Martian Day (Sol) 12 | 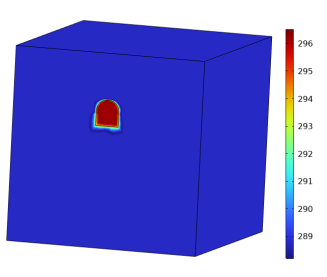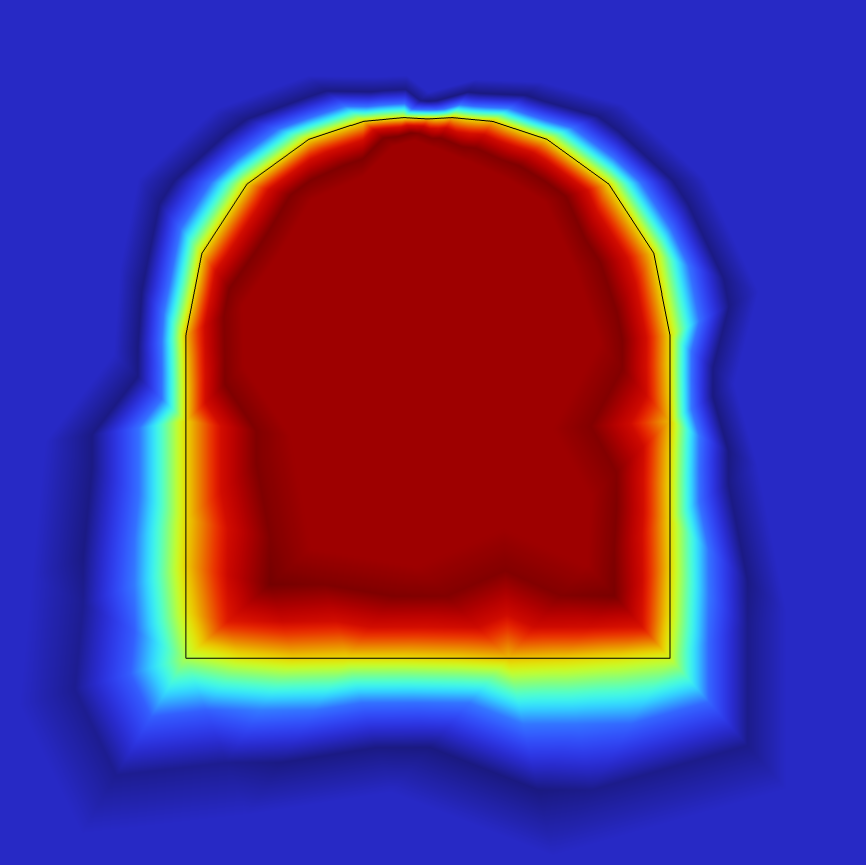 | 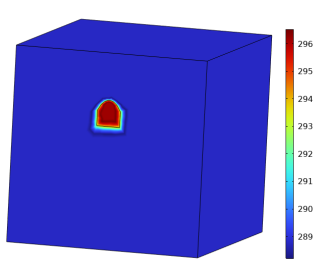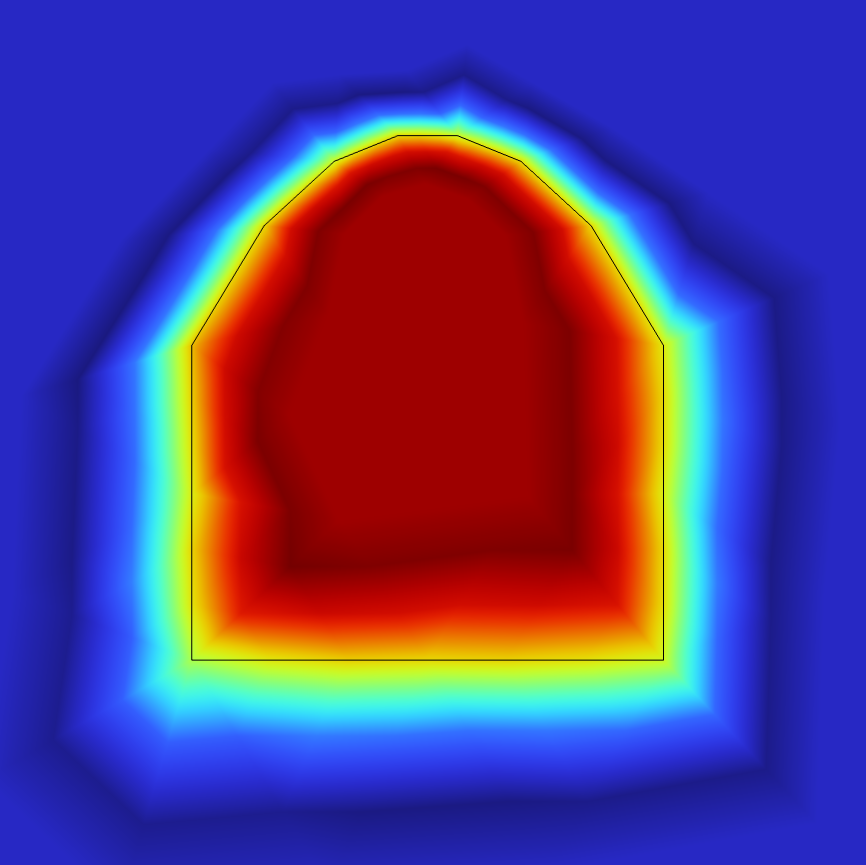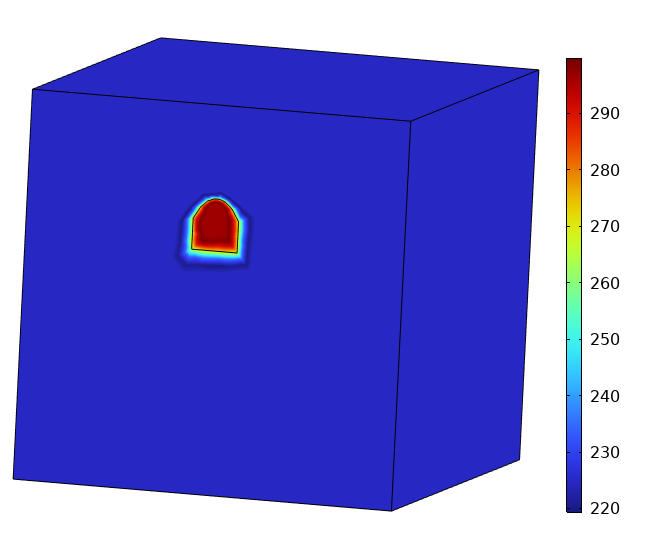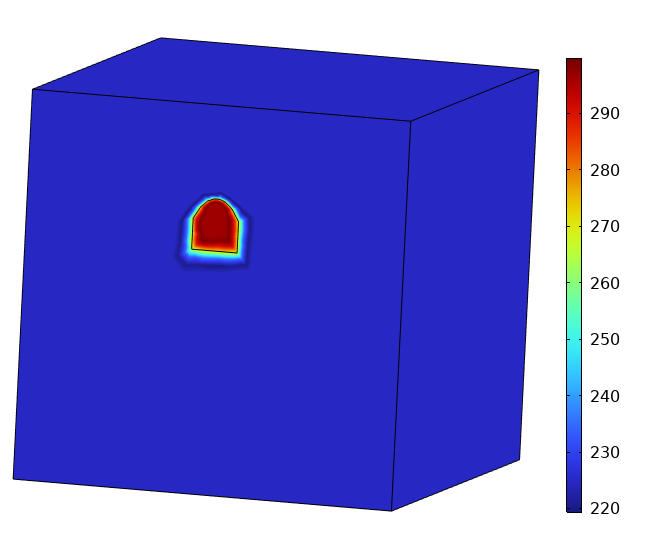 | 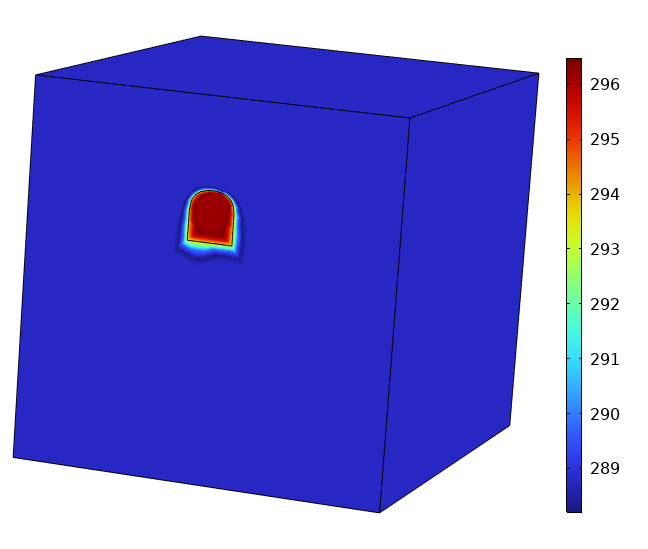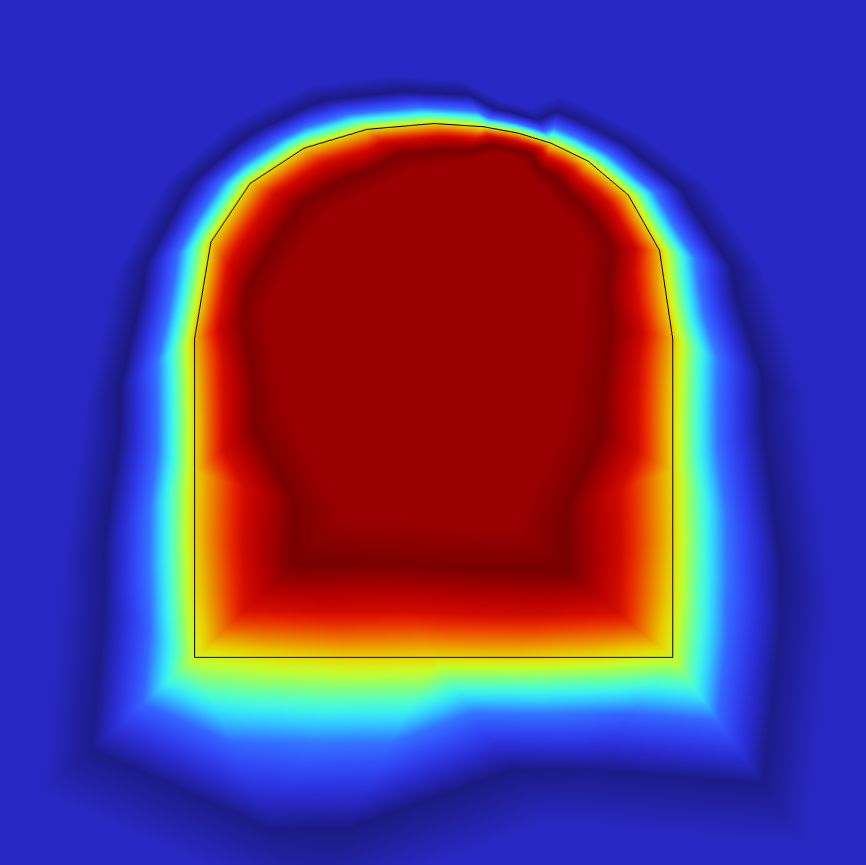 |
| Martian Day (Sol) 16 | 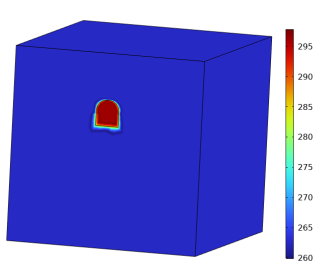 |  |  |
| Martian Day (Sol) 20 |  |  |  |

**Supplementary Table 5 Summary of temperature field distributions and detailed diagrams for eggshell arches using different wall materials (Ls=270°)**

|  | **Eggshell arch (Aerogel)** | **Eggshell arch (Fiber polymer)** | **Eggshell arch (Basalt)** |
| --- | --- | --- | --- |
| Martian Day (Sol) 0 |  |  |  |
| Martian Day (Sol) 4 |  |  |  |
| Martian Day (Sol) 8 |  |  |  |

**Continued Table 5**

|  | **Eggshell arch (Aerogel)** | **Eggshell arch (Fiber polymer)** | **Eggshell arch (Basalt)** |
| --- | --- | --- | --- |
| Martian Day (Sol) 12 |  |  |  |
| Martian Day (Sol) 16 |  |  |  |
| Martian Day (Sol) 20 |  |  |  |

**Supplementary Table 6 Summary of temperature field distributions and detailed diagrams for eggshell arches using different wall materials (Ls=90°)**

|  | **Eggshell arch (Aerogel)** | **Eggshell arch (Fiber polymer)** | **Eggshell arch (Basalt)** |
| --- | --- | --- | --- |
| Martian Day (Sol) 0 |  |  |  |
| Martian Day (Sol) 4 |  |  |  |
| Martian Day (Sol) 8 |  |  |  |

**Continued Table 6**

|  | **Eggshell arch (Aerogel)** | **Eggshell arch (Fiber polymer)** | **Eggshell arch (Basalt)** |
| --- | --- | --- | --- |
| Martian Day (Sol) 12 |  |  |  |
| Martian Day (Sol) 16 |  |  |  |
| Martian Day (Sol) 20 |  |  |  |

**Supplementary Table 7 Summary of temperature field distributions and detailed diagrams for eggshell arches using different wall materials (General)**

|  | **Eggshell arch (Aerogel)** | **Eggshell arch (Fiber polymer)** | **Eggshell arch (Basalt)** |
| --- | --- | --- | --- |
| Martian Day (Sol) 0 |  |  |  |
| Martian Day (Sol) 4 |  |  |  |
| Martian Day (Sol) 8 |  |  |  |

**Continued Table 7**

|  | **Eggshell arch (Aerogel)** | **Eggshell arch (Fiber polymer)** | **Eggshell arch (Basalt)** |
| --- | --- | --- | --- |
| Martian Day (Sol) 12 |  |  |  |
| Martian Day (Sol) 16 |  |  |  |
| Martian Day (Sol) 20 |  |  |  |

**Supplementary Data 4 Summary of the effect of arch span on stability (varying parameter B)**

| **Arch span/m** | **Displacement on x-axis/mm** | | | | | **Displacement on y-axis/mm** | | | | | **Minimum principal compressive stress/kPa** | | | | |
| --- | --- | --- | --- | --- | --- | --- | --- | --- | --- | --- | --- | --- | --- | --- | --- |
|  | **Point 1** | **Point 2** | **Point 3** | **Point 4** | **Point 5** | **Point 1** | **Point 2** | **Point 3** | **Point 4** | **Point 5** | **Point 1** | **Point 2** | **Point 3** | **Point 4** | **Point 5** |
| 2.8 | 0.113 | 2.78 | 2.08 | 0.56 | 0. 013 | -16.55 | -18.74 | -19.67 | -21.07 | -21.95 | -150.12 | -33.16 | -34.60 | -34.74 | 0.145 |
| 2.9 | 0.157 | 2.91 | 2.19 | 1.01 | 0. 015 | -16.37 | -18.76 | -19.70 | -21.16 | -22.05 | -148.72 | -33.21 | -34.72 | -34.65 | 0.125 |
| 3.0 | 0. 119 | 2.99 | 2.26 | 1.01 | 0. 015 | -16.36 | -18.77 | -19.73 | -21.24 | -22.15 | -152.82 | -33.25 | -34.76 | -34.89 | 0.134 |
| 3.1 | 0.120 | 3.06 | 2.32 | 1.02 | 0.017 | -16.32 | -18.79 | -19.76 | -21.32 | -22.25 | -154.24 | -33.28 | -34.85 | -34.93 | 0.139 |
| 3.2 | 0.116 | 3.15 | 2.38 | 1.02 | 0.013 | -16.29 | -18.81 | -19.79 | -21.41 | -22.36 | -150.22 | -33.28 | -34.83 | -35.14 | 0.146 |
| 3.3 | 0.145 | 3.22 | 2.45 | 1.03 | 0.014 | -16.25 | -18.83 | -19.83 | -21.50 | -22.46 | -153.07 | -33.30 | -34.95 | -34.27 | 0.145 |
| 3.4 | 0.115 | 3.30 | 2.52 | 1.03 | 0.014 | -16.24 | -18.86 | -19.86 | -21.59 | -22.57 | -151.21 | -33.35 | -34.99 | -34.87 | 0.170 |

**Supplementary Data 5 Summary of the effect of arch span on thermal performance**

| **Heat loss/W** | **Arch span:2.8m** | **Arch span:2.9m** | **Arch span:3.0m** | **Arch span:3.1m** | **Arch span:3.2m** | **Arch span:3.3m** | **Arch span:3.4m** |
| --- | --- | --- | --- | --- | --- | --- | --- |
| Mars midnight | 267.09 | 277.50 | 273.63 | 283.65 | 285.35 | 289.65 | 290.98 |
| Mars noontime | 18.31 | 19.03 | 18.76 | 19.45 | 19.53 | 19.85 | 19.93 |

**Supplementary Data 6 Summary of the effect of arch height on stability (varying L, w: 0.65)**

| **Arch**  **height/m** | **Displacement on x-axis/mm** | | | | | **Displacement on y-axis/mm** | | | | | **Minimum principal compressive stress/kPa** | | | | |
| --- | --- | --- | --- | --- | --- | --- | --- | --- | --- | --- | --- | --- | --- | --- | --- |
|  | **Point 1** | **Point 2** | **Point 3** | **Point 4** | **Point 5** | **Point 1** | **Point 2** | **Point 3** | **Point 4** | **Point 5** | **Point 1** | **Point 2** | **Point 3** | **Point 4** | **Point 5** |
| 1.05 | 0. 098 | 2.67 | 1.99 | 0.35 | 0. 014 | -16.49 | -18.94 | -19.92 | -21.56 | -22.14 | -144.40 | -33.25 | -35.04 | -35.45 | 0.087 |
| 1.15 | 0. 113 | 2.78 | 2.08 | 0.56 | 0. 013 | -16.55 | -18.89 | -19.86 | -21.46 | -22.15 | -147.08 | -33.26 | -35.02 | -35.15 | 0.100 |
| 1.25 | 0. 108 | 2.88 | 2.17 | 0.79 | 0. 013 | -16.44 | -18.83 | -19.79 | -21.36 | -22.15 | -151.25 | -33.25 | -34.81 | -35.10 | 0.130 |
| 1.35 | 0. 119 | 2.99 | 2.26 | 1.01 | 0. 015 | -16.36 | -18.77 | -19.73 | -21.24 | -22.15 | -152.82 | -33.25 | -34.76 | -34.89 | 0.134 |
| 1.45 | 0. 131 | 3.10 | 2.35 | 1.22 | 0. 015 | -16.26 | -18.72 | -19.67 | -21.12 | -22.15 | -151.06 | -33.19 | -34.62 | -34.60 | 0.184 |
| 1.55 | 0.143 | 3.22 | 2.45 | 1.42 | 0.015 | -16.16 | -18.67 | -19.61 | -21.00 | -22.16 | -152.32 | -33.21 | -34.60 | -34.39 | 0.237 |
| 1.65 | 0.155 | 3.35 | 2.56 | 1.61 | 0.015 | -16.06 | -18.61 | -19.55 | -20.88 | -22.17 | -153.17 | -33.19 | -34.51 | -34.39 | 0.218 |

**Supplementary Data 7 Summary of the effect of arch height on thermal performance (varying L, w: 0.65)**

| **Heat loss/W** | **Arch height:1.05m** | **Arch height:1.15m** | **Arch height:1.25m** | **Arch height:1.35m** | **Arch height:1.45m** | **Arch height:1.55m** | **Arch height:1.65m** |
| --- | --- | --- | --- | --- | --- | --- | --- |
| Mars midnight | 272.44 | 271.12 | 278.52 | 273.63 | 286.61 | 295.00 | 297.90 |
| Mars noontime | 18.67 | 18.61 | 19.09 | 18.76 | 19.64 | 20.22 | 20.42 |

**Supplementary Data 8 Summary of the effect of arch height on stability (varying w, L: 4)**

| **Arch**  **height/m** | **Displacement on x-axis/mm** | | | | | **Displacement on y-axis/mm** | | | | | **Minimum principal compressive stress/kPa** | | | | |
| --- | --- | --- | --- | --- | --- | --- | --- | --- | --- | --- | --- | --- | --- | --- | --- |
|  | **Point 1** | **Point 2** | **Point 3** | **Point 4** | **Point 5** | **Point 1** | **Point 2** | **Point 3** | **Point 4** | **Point 5** | **Point 1** | **Point 2** | **Point 3** | **Point 4** | **Point 5** |
| 1.15 | 0. 130 | 3.02 | 2.34 | 1.29 | 0. 014 | -16.34 | -18.78 | -19.61 | -21.08 | -22.22 | -153.91 | -33.56 | -30.98 | -34.41 | 0.141 |
| 1.25 | 0. 127 | 3.00 | 2.29 | 1.34 | 0. 019 | -16.35 | -18.77 | -19.66 | -21.17 | -22.18 | -151.57 | -33.32 | -33.51 | -34.61 | 0.133 |
| 1.35 | 0. 119 | 3.00 | 2.26 | 1.01 | 0. 015 | -16.36 | -18.77 | -19.73 | -21.24 | -22.15 | -152.82 | -33.25 | -34.76 | -34.89 | 0.134 |
| 1.45 | 0. 122 | 2.97 | 2.22 | 0.91 | 0. 016 | -16.37 | -18.79 | -19.80 | -21.28 | -22.11 | -153.39 | -33.32 | -35.60 | -34.88 | 0.172 |
| 1.55 | 0.120 | 2.95 | 2.18 | 0.83 | 0.015 | -16.37 | -18.81 | -19.88 | -21.32 | -22.08 | -147.34 | -33.62 | -36.23 | -34.72 | 0.168 |
| 1.65 | 0.121 | 2.93 | 2.13 | 0.77 | 0.015 | -16.38 | -18.83 | -19.96 | -21.34 | -22.04 | -152.18 | -34.03 | -37.34 | -34.71 | 0.125 |

**Supplementary Data 9 Summary of the effect of arch height on thermal performance (varying w, L: 4)**

| **Heat loss/W** | **Arch height:1.15m** | **Arch height:1.25m** | **Arch height:1.35m** | **Arch height:1.45m** | **Arch height:1.55m** | **Arch height:1.65m** |
| --- | --- | --- | --- | --- | --- | --- |
| Mars midnight | 279.95 | 282.50 | 273.63 | 292.27 | 296.62 | 301.93 |
| Mars noontime | 19.16 | 19.35 | 18.76 | 20.03 | 20.36 | 20.07 |
